# Functional redundancy of division specific penicillin‐binding proteins in *Bacillus subtilis*

**DOI:** 10.1111/mmi.13765

**Published:** 2017-08-29

**Authors:** Jad Sassine, Meizhu Xu, Karzan R. Sidiq, Robyn Emmins, Jeff Errington, Richard A. Daniel

**Affiliations:** ^1^ Centre for Bacterial Cell Biology, Institute for Cell and Molecular Biosciences, The Medical School Newcastle University Newcastle upon Tyne NE2 4AH UK; ^2^ Present address: Xiamen Ampearl Jewellery Ltd., 24A No.327 Pacific Square, Jiahe Road, Siming District Xiamen 361012 P.R. China; ^3^ Present address: General Sciences Department Charmo University 46023 Chamachamal Sulaimani Kurdistan Region Iraq; ^4^ Present address: GSK‐UK, New Frontiers Science Park, Third Avenue Harlow Essex CM19 5AW UK

## Abstract

Bacterial cell division involves the dynamic assembly of a diverse set of proteins that coordinate the invagination of the cell membrane and synthesis of cell wall material to create the new cell poles of the separated daughter cells. Penicillin‐binding protein PBP 2B is a key cell division protein in *Bacillus subtilis* proposed to have a specific catalytic role in septal wall synthesis. Unexpectedly, we find that a catalytically inactive mutant of PBP 2B supports cell division, but in this background the normally dispensable PBP 3 becomes essential. Phenotypic analysis of *pbpC* mutants (encoding PBP 3) shows that PBP 2B has a crucial structural role in assembly of the division complex, independent of catalysis, and that its biochemical activity in septum formation can be provided by PBP 3. Bioinformatic analysis revealed a close sequence relationship between PBP 3 and *Staphylococcus aureus* PBP 2A, which is responsible for methicillin resistance. These findings suggest that mechanisms for rescuing cell division when the biochemical activity of PBP 2B is perturbed evolved prior to the clinical use of β‐lactams.

## Introduction

*Bacillus subtilis* exhibits moderate resistance to a variety of antimicrobial compounds, particularly those that target cell wall biosynthesis (e.g., β‐lactams, nisin and cationic antimicrobial peptides) (Helmann, 2006). The integrity of the cell wall is crucial for the viability of bacteria because it protects the cell from mechanical damage derived either from environmental factors or the osmotic pressure of the cytoplasm, which would otherwise burst the cell membrane and cause cell lysis. The major structural component of most bacterial cell walls is a net like matrix of long glycan strands cross‐linked by peptide bridges (peptidoglycan; PG) (Sobhanifar *et al*., 2013). During cell growth, and also during cell division, new cell wall material is synthesised to allow cell expansion and to make the dividing wall (septum). The final steps of PG synthesis are presumed to be carried out predominantly by bifunctional (class A) penicillin‐binding proteins (PBPs) that possess both glycosyltransferase activity, used to extend the glycan chains, and transpeptidase (TPase) activity, which generates the peptide cross‐links. Additional monofunctional (class B) PBPs that have only TPase activity are also present and have essential roles in PG synthesis, although their precise biochemical role is unclear. β‐lactam antibiotics inhibit the TPase activity of PBPs with varying degrees of specificity (Spratt, 1975). Previous analyses have indicated that resistance/tolerance to β‐lactam antibiotics is mediated by transcriptional regulation through extra cytoplasmic function (ECF) sigma factors and the messenger molecule c‐di‐AMP (Luo and Helmann, 2012; Commichau *et al*., 2015). Full details of these resistance mechanisms remain to be characterised.

Bacterial genomes often encode 10 or more PBPs, although many of them are non‐essential, suggesting functional redundancy. However, there is usually at least one essential PBP and several laboratories have shown, in various organisms, that a PBP specialised for wall synthesis in the division septum is essential (Yanouri *et al*., 1993; Kato *et al*., 1988; Massidda *et al*., 1998; Daniel *et al*. 2000; Datta *et al*., 2006; Sauvage *et al*., 2014). In *B. subtilis*, the essential division specific enzyme, PBP 2B, is targeted to division sites by interaction with one or more components of the division machinery, orchestrated by the FtsZ protein (Daniel *et al*., 2000 *;* 2006). The functions of the other *B. subtilis* PBPs in cell growth are less well understood, although in *B. subtilis* it seems that PBP 2A has a major role in elongation of the cylindrical part of the wall, albeit a role that is partially redundant to that of PBP H (Wei *et al*., 2003).

All PBPs carry a well‐characterised catalytic triad with a conserved active site serine. This serine makes a covalent adduct with β‐lactam antibiotics, which renders the enzyme inactive. Substitutions of this serine completely inactivate the TPase activity of the enzyme (Goffin and Ghuysen, 2002). Remarkably, we have found that elimination of catalytic activity by substitution of the active site serine of PBP 2B (PBP 2B^(S309A)^) has almost no effect on cell division in *B. subtilis*, even though depletion of the entire protein is lethal (Daniel *et al*., 1996). The structural role of PBP 2B and its equivalents has been quite well‐documented in *B. subtilis* (Daniel *et al*., 2006) and in other bacteria (Goehring and Beckwith, 2005; Karimova *et al*., 2005; Wissel *et al*., 2005; Datta *et al*., 2006; Valbuena *et al*., 2006), but the notion that catalytic activity was not essential was quite unexpected. Further analysis of the PBP 2B^(S309A)^ mutant revealed that PBP 3, previously shown to be dispensable (Murray *et al*., 1996), takes on an essential role in this background. We show that the essential function of PBP 3, in the absence of biochemically active PBP 2B, lies in its TPase activity.

By characterising the sensitivity of *B. subtilis* strains lacking individual PBPs, we have found that the loss of PBP 3 or PBP 2A makes *B. subtilis* significantly more sensitive to β‐lactams. The increased sensitivity of the PBP 2A null mutant is potentially explained by the fact that the mutant does exhibit a mild growth defect (Murray *et al*., 1998), but was unexpected for the strain lacking PBP 3. Overall, our results suggest that the division machinery is assembled in such a way that if the TPase activity of the architectural PBP 2B enzyme is inactivated (e.g., by covalent antibiotic binding), PBP 3 can provide the necessary activity to allow the division septum to be synthesised. The results also indicate that PBP 3 is intrinsically resistant to the binding of several β‐lactams, suggesting that it may provide a resistance mechanism comparable to that recently acquired by *Staphylococcus aureus* MRSA, a notion further supported by sequence analysis. This may explain how the acquisition of a heterologous resistant PBP can provide antibiotic resistance without the immediate need for extensive protein‐protein interactions with the resident synthetic machinery.

## Results

### A mutant with biochemically inactive PBP 2B is viable

**Figure 1:**
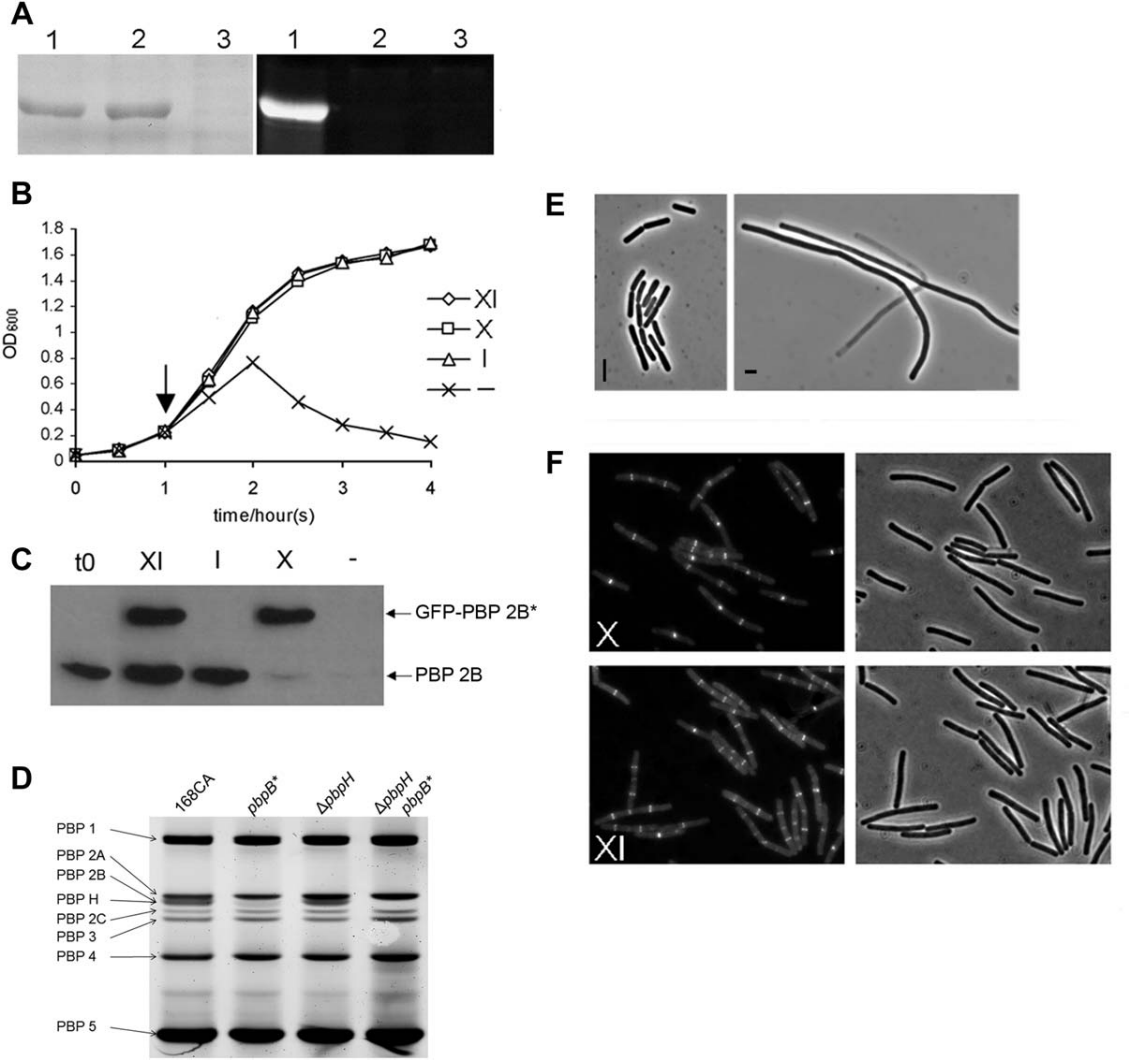
A. Penicillin binding activity of wild type and mutant forms of PBP 2B. PBP 2B and PBP 2B^(S309A)^ were overproduced in *E. coli* and the membrane fraction was purified, labelled with Bocillin‐FL and separated by SDS‐PAGE. The left panel is an image of the protein gel following Coomassie staining showing that similar amounts of total protein and overproduced PBP 2B protein were present on the gel and the right panel shows an image of the same gel when scanned for fluorescence. The gel was loaded with membrane preparations from *E. coli* over expressing WT *pbpB* (lane 1); pbpB* (2); control (vector only) (3). B. Growth curve of strain 4004 in various inducer conditions. Strain 4004, which contains the pbpB* allele controlled by a xylose induced promoter (P_*xyl*_) and a WT *pbpB* controlled by an IPTG induced promoter (P_*spac*_), was grown in PAB with IPTG then transferred to Fresh PAB media with various combinations of supplements (XI, media supplemented with xylose and IPTG; X, xylose alone; I, IPTG alone; ‐, no addition.) and the growth against time was measured by optical density. C. Inducer dependence of PBP 2B and GFP‐PBP 2B. Western blot of total protein samples taken at the start of the experiment (t0) and 1 h after removing or adding inducers (arrow in panel B) and probed with polyclonal antisera specific for PBP 2B. D. An image obtained by fluorography showing the binding profile of bocillin–FL to *B. subtilis* PBPs from strains 168 (wild‐type), pbpB*, Δ*pbpH* and ΔpbpH pbpB*. The identity of each PBP is indicated on the left of the image. E. Phase contrast images of strain 4004 grown in the presence of IPTG (I) or in the absence of any inducers (‐). F. Localisation of GFP‐PBP 2B^(S309A)^ in cells in the presence or absence of WT *pbpB* expression. Cells of strain 4004 grown with xylose and IPTG (XI) and xylose alone (X). Each panel shows a fluorescence micrograph and the corresponding phase contrast image.

During our work to characterise the essential cell division gene *pbpB*, coding for PBP 2B, we tested whether the transpeptidase activity of the protein was important for its function in synthesising the division septum. We aligned PBP 2B with PBP 2X of *S. pneumoniae*, and other PBPs that have defined active sites, to identify the probable active site residue required for transpeptidation (Pares *et al*., 1996; summarised in Supporting Information Fig. S1 panel A). From this analysis, the serine at position 309 was clearly located in the consensus sequence for the active site of these PBPs. The amino acid numbering for PBP 2B used here is based on the translational start site being located at the fourth amino acid of the coding sequence as it is currently annotated in Swiss‐Prot entry PBPB_BACSU Q07868 (Xu, 2008). We then made a serine to alanine (S309A) substitution by site directed mutagenesis to generate a mutant *pbpB* gene denoted pbpB*. To confirm that the S309A mutation had inactivated the TPase activity of the protein, both the wild type and the mutant forms of PBP 2B were overexpressed in *E. coli*. Both proteins were found to be predominantly present in the membrane fraction of *E. coli*, but on exposure of the proteins to a fluorescent derivative of a β‐lactam antibiotics, bocillin‐FL, the mutant protein did not detectably bind bocillin, whereas the wild type protein was heavily labelled (Fig. 1A lane 1; compared to lane 2), confirming that the S309 residue is required for penicillin binding.

The same pbpB* mutation was then introduced into *B. subtilis* at the ectopic *amyE* locus under the control of a xylose‐inducible promoter (P_*xyl*_). The coding sequence of the green fluorescent protein (*gfp*) gene was also fused to the N‐terminal coding end of the pbpB* gene so that the localisation of the mutant protein could be studied. Then, a P_*spac*_ (IPTG‐dependent) promoter was inserted in front of the wild‐type copy of *pbpB*, to allow repression of the native copy of the gene. Unexpectedly, in the absence of IPTG but in the presence of xylose, thus expressing only the mutant protein, the strain (4004) was found to grow as well as that expressing the wild‐type protein (Fig. 1B).

Fluorescence microscopy of the cells showed that the mutant protein was targeted to division sites, similar to the wild type protein, and the cells were morphologically normal even when only the mutant copy of *pbpB* was expressed (Fig. 1F, panel X). However, when xylose and IPTG were both withheld, repressing both copies of *pbpB*, growth of the culture was severely impaired (Fig. 1B). Microscopic examination of these cultures showed that the cells grew as long filaments which eventually lysed (Fig. 1E), consistent with the previous result that PBP 2B is essential for cell division. These results suggested that PBP 2B^(S309A)^ was still functional for cell division, although it was possible that the P_*spac*_ promoter was not sufficiently repressed and provided sufficient wild‐type PBP 2B for division to occur. Western blotting using polyclonal anti‐PBP 2B antisera (Fig. 1C) indicated the presence of a very small amount of wild‐type PBP 2B in total protein samples of strain 4004 grown in the absence of IPTG (Fig. 1C lane ‘X’). However, a similar amount of PBP 2B was also detectable when strain 4004 was grown in the absence of both IPTG and xylose (Fig. 1C lane ‘‐’), although under these conditions division was not well supported (as determined by microscopy; Fig. 1E).

To eliminate the possibility that leaky transcription from the P_*spac*_ promoter was providing sufficient wild‐type PBP 2B to allow cell division/growth, and to confirm that PBP 2B^(S309A)^ could support cell division, we directly replaced the wild‐type *pbpB* allele with the mutant allele to generate a strain that was isogenic with the wild type except for the presence of the pbpB* mutation. The growth rate of this mutant strain (4001) was again similar to that of the wild‐type strain 168, while microscopic analysis showed that the average cell length of the mutant cells (2.4 ± 0.4 µm) was slightly (about 25%) greater than that of the wild type (1.9 ± 0.3 μm) (Table 1). To confirm that the mutant protein had lost its TPase activity and was unable to bind bocillin when expressed in *B. subtilis*, live cell bocillin‐FL labelling was used for both the wild‐type strain 168 and the mutant strain 4001 (Fig. 1D). As PBP 2B co‐migrated with PBP H in the gel, Δ*pbpH* and ΔpbpH pbpB* mutants (strains 4017 and 4024 respectively) were constructed and analysed by live cell bocillin‐FL labelling to confirm the lack of labelling of the mutant PBP 2B. These results confirmed that while PBP 2B protein is absolutely required for cell division, its TPase activity is not.

**Table 1:** Cell length analysis.

Strains (relevant genotype)	Mean cell length^a^ µm ± SD		Mean cell width^a^ µm ± SD^a^	
	– IPTG	+ IPTG	– IPTG	+ IPTG
168CA (wild type)	1.9 ± 0.3		0.8 ± 0.08	
4001 (*pbpB* ^*^)	2.4 ± 0.4	ND	0.8 ± 0.07	ND
KS50 (P*_*spac*_ pbpC*)	1.8 ± 0.4	1.8 ± 0.4	0.7 ± 0.09	0.7 ± 0.1
KS51 (*pbpB* ^*^ P*_*spac*_ pbpC*)	2.4 ± 0.7	2.5 ± 0.6	0.7 ± 0.1	0.7 ± 0.1
KS53(P*_*spac*_ pbpC* ^*^)	1.8 ± 0.4	1.8 ± 0.4	0.8 ± 0.1	0.7 ± 0.08
KS54 (*pbpB* ^*^ P*_*spac*_ pbpC* ^*^)	2.4 ± 0.3	2.4 ± 0.4	0.8 ± 0.09	0.8 ± 0.07
KS52 (P*_*spac*_ pbpC ΔpbpC(cat)*)	1.8 ± 0.3	1.8 ± 0.3	0.8 ± 0.07	0.8 ± 0.07

**a.** Dimensions were determined from images of exponentially growing cultures in which the cells were stained with membrane dye (FM 5.95). Greater than 100 cell measurements were taken for each sample; the values shown represent the mean cell size and the standard deviation (SD) for that sample.

ND indicates where cell length was not determined.

### Biochemically inactive PBP 2B mutant requires the function of PBP 3

The unexpected result that a mutant carrying a biochemically inactive form of PBP 2B was viable suggested that either cross‐linking of glycan chains is not required at the division site or that this activity can be provided by another enzyme. To test the latter idea, mutations in each of the known vegetatively expressed PBP genes (*pbpA*, *pbpC, pbpD, pbpF, pbpH* and *ponA*) were introduced into both the pbpB* mutant and a wild‐type strain, with the expectation that knocking out a gene that could substitute for the cell division TPase activity of PBP 2B would be lethal in the pbpB* mutant background. As expected, in the *pbpB* ^+^ background introduction of any of the null mutations gave colonies at high frequency expected for single uncomplicated transformation events (Rivolta and Pagni, 1999). Similar transformation results were obtained for the pbpB* mutant with all of the null mutations except *pbpC*. For *pbpC*, the transformation efficiency was much lower (less than 10% of that seen for the wild‐type strain) and two colonies types were obtained; the majority were small and could not in general be sub cultured, whereas a minority were normal in size. Microscopic examination of the small colonies revealed that their cells were filamentous, whereas those of the large colonies were wild‐type. Sequencing of the *pbpB* locus from several of the large and a few small colonies that grew up showed that what was grown had lost the pbpB* mutation. These were most likely generated by a double transformation event in which they acquired the unselected copy of the wild‐type allele of *pbpB* together with the *pbpC* null mutation. These results suggested that PBP 3 is essential in the absence of the TPase activity of PBP 2B.

To test whether the TPase activity of PBP 3 was required for complementation of PBP 2B^(S309A)^, and to eliminate the possibility that the *pbpC* null mutation had unexpected polar effects on neighbouring gene expression, we constructed a plasmid carrying a mutant pbpC* allele (PBP 3^(S410A)^). This mutation was expected to eliminate its TPase activity as it removed the serine residue that was predicted to be located in the active site of the PBP (Supporting Information Fig. S1A). This plasmid (pSG5666) was then integrated into the chromosome at the *pbpC* gene locus. In a wild‐type recipient, sequence analysis of 20 independent clones revealed that about 75% of the clones picked up the mutant allele in the functional copy of *pbpC* a frequency close to expectation, based on a single crossover recombination event. However, none of the pbpB* recipients (0/12 checked by sequencing) acquired the pbpC* mutation. Thus, the pbpC* mutation probably renders PBP 3 unable to complement the function of PBP 2B^(S309A)^. To confirm this, a strain (4009) was constructed with both the pbpC* and pbpB* mutant alleles and a second wild‐type copy of *gfp‐pbpB* under the control of the P*_xyl_* promoter. In the presence of xylose, to allow expression of the catalytically active version of *pbpB*, strain 4009 was indistinguishable from the wild‐type, but in the absence of xylose the cells became filamentous and could not be cultured. These results indicate that the TPase activity of either PBP 2B or PBP 3 is essential for cell division, and this activity is either unique to these PBPs or is related to how these PBPs interact with other division proteins.

### Mid cell localisation of PBP 3

**Figure 2:**
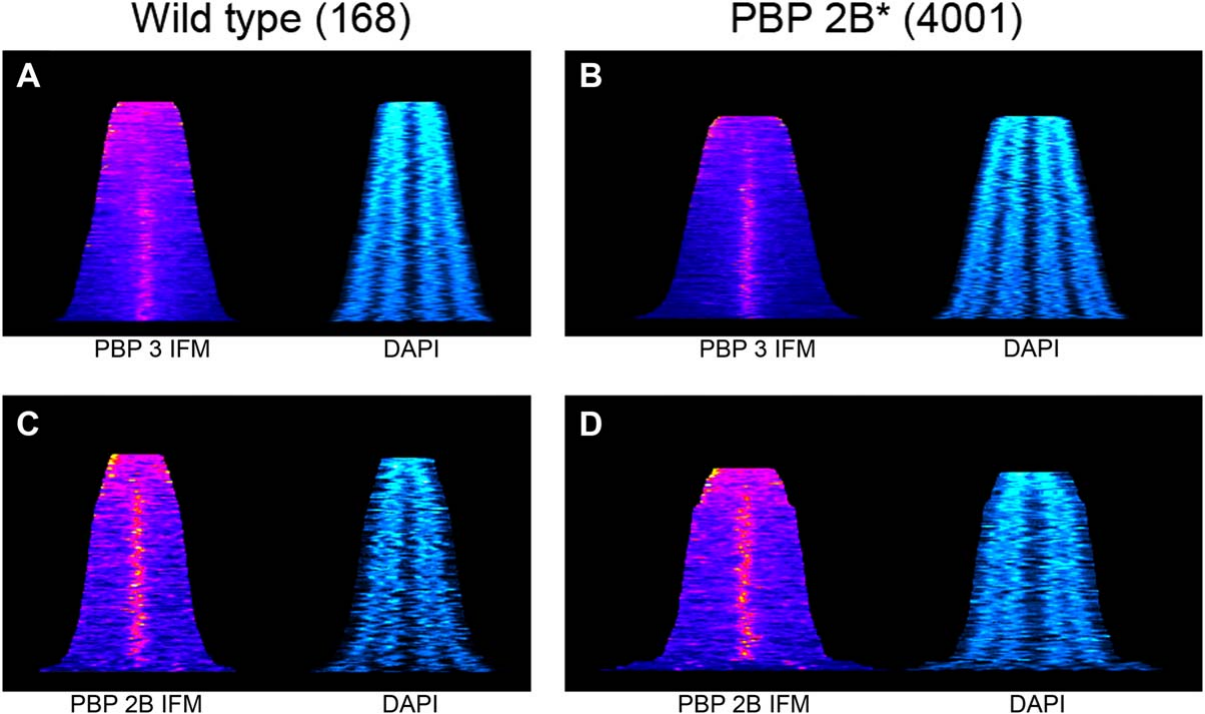
Graphical representations of the subcellular distribution of PBP 3 and PBP 2B as a function of cell length. To permit the representation of the distribution of PBP 3 and PBP 2B on the long axis of the cell in a population of cells, immunofluorescence images were analysed using a line scan function. The output of this was then used to generate heat maps where each horizontal line represents an individual cell (sorted according to cell length) and the colour indicates the level of fluorescence detected (ranging from dark blue to red and yellow in terms of strength). To provide a second landmark for the interpretation of the images, the subcellular distribution of the chromosomal DNA was determined exploiting DAPI labelling and quantitation of the fluorescence signal in the same way. Panels **A** and **B** show PBP 3 cellular distribution in the wild‐type strain and 4001 (pbpB*) respectively, whereas **C** and **D** indicate PBP 2Bs distribution. For each immunofluorescent analysis, the corresponding DNA distribution is shown. For 168, the data represents the analysis of > 140 cells, whereas for 4001 the panel is the summary of > 400 cells.

As described earlier, the strain with an inactive PBP 2B (4001), although growing at a comparable rate to the wild‐type strain, exhibited elongated cells during exponential growth (∼25% longer; Table 1). Thus, although PBP 3 activity can support cell division it is apparently not as efficient as PBP 2B. This suggested that PBP 3 may not be recruited to the division site efficiently and so is not able to provide the required TPase activity to permit the normal progression of cell division in the pbpB* mutant. To test this, we initially used the P*_xyl_* inducible GFP‐PBP 3 described by (Scheffers *et al*., 2004). This was found to be at least partly functional, as it supported cell division in the absence of active PBP 2B (strain 4005). However, Western blot analysis using a polyclonal anti‐PBP3 antibody showed that the GFP fusion was either unstable, or that the expressed protein was processed such that detectable levels of both PBP 3 and GFP‐PBP 3 were present in the culture, even when chloramphenicol selection was maintained during growth (Supporting Information Fig. S2 A). Consequently, we used immunofluorescence to resolve the localisation question. Initial inspection of images of cells suggested that PBP 3 may exhibit a bias toward localising to the division site and the poles of the cell in strain lacking an active PBP 2B compared to the wild type, but it was difficult to visually quantify any significant differences between strains where PBP 2B was functional or inactive (Supporting Information Fig. S2 B and C). To obtain a qualitative understanding of the sub‐cellular distribution of PBP 3, heat maps representing the fluorescent signal obtained by IFM for the long axis of individual cells were generated for both wild type (168) and pbpB* (4001) strains (Fig. 2A and B). PBP 3 tended to localise mainly at mid cell, except in short cells in which a more distributed or polar localization was evident. The pattern was similar in wt and pbpB* cells except that the mutant cells were in general longer and almost all cells had PBP 3 enriched at mid cell position in elongated cell. The PBP 2B distribution was similar in both wild‐type and mutant, with a distinct mid cell localization except in a roughly similar proportion of the short cells. For comparison, the same analysis was done using antisera specific for PBP 2B (Fig. 2C and D), where clear accumulation of the protein occurs at the mid cell position even in relatively short cells, suggesting earlier localisation.

The image analysis also showed that the cells of strains lacking PBP 2B were clearly longer than those of the wild‐type, suggesting, perhaps, an insufficiency of PBP3. However, overexpression of the *pbpC* from the strong hyperspank promoter (Vavrová *et al*., 2010) did not change cell length or morphology in a pbpB* background (Supporting Information Fig. S3 and Table 1).

### PBP 3 localisation at division sites depends on FtsZ and PBP 2B

**Figure 3:**
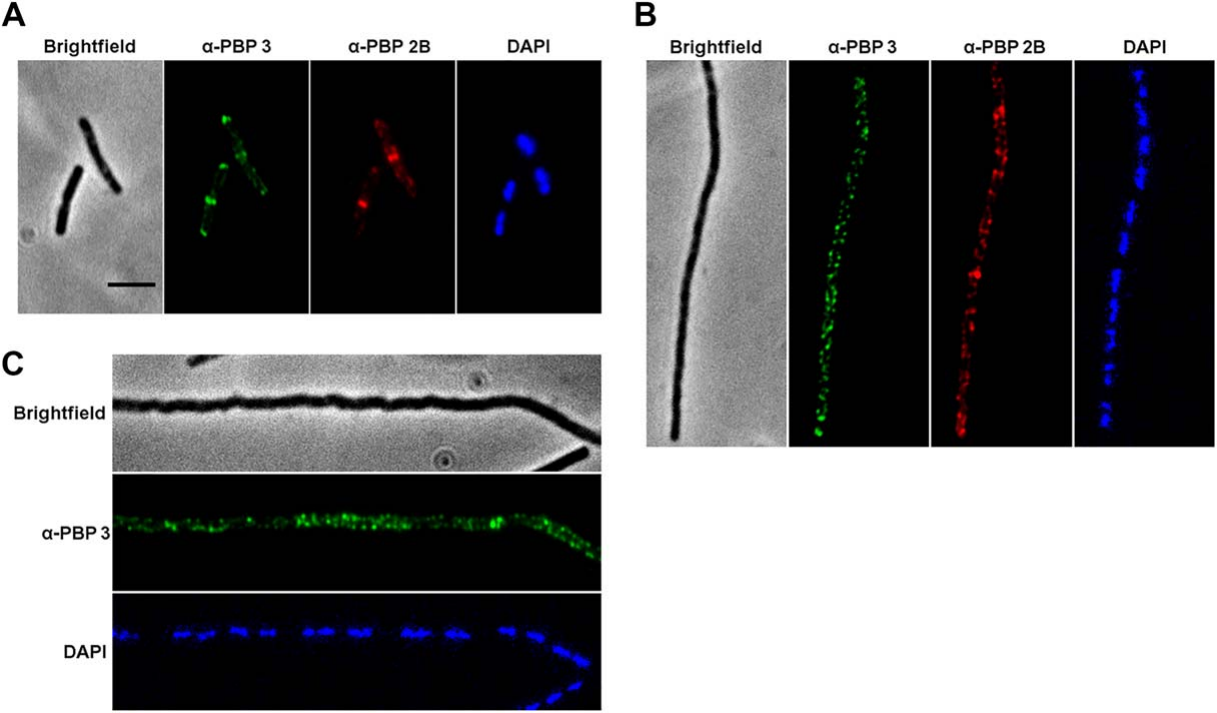
Cellular localisation of PBP 3 in FtsZ and PBP 2B depleted strains. Epifluorescence images of cells immunolabeled for PBP 2B and/or PBP 3 for the wild type (strain 168, A, a strain depleted for FtsZ (strain 1801, B) and a strain depleted for PBP 2B (strain 3941, C). Each panel shows the phase contrast image of the cells visualised, epifluorescence images corresponding to the specific immune stains used and finally a DAPI stain images to show the localisation of the DNA.

Assembly of the divisome is regulated by the polymerization of FtsZ, a tubulin like protein, into a ring at midcell (Bi and Lutkenhaus, 1991). Depletion of FtsZ resulted in a shift in the localization of PBP 2B from midcell to the lateral wall and an arrest in cell division (Scheffers *et al*., 2004). The depletion of PBP 2B also results in a cell division block, which suggests that PBP 2B might have a role in the co‐assembly of other cell division proteins (Daniel *et al*., 2006). To investigate whether PBP 3 is part of the multiprotein complex involved in PG synthesis during cell division, we examined the localization of PBP 3 in PBP 2B or FtsZ depleted cells using IFM. Immediately after the washing step to begin depletion of PBP 2B, PBP 3 showed the expected predominant localization at midcell and the cell poles, with only occasional localization along the cell periphery (Fig. 3A). One hour after the removal of inducers, the cells were filamentous as expected following the depletion of FtsZ or PBP 2B. In both cases, PBP 3 localized in a dispersed peripheral pattern with no sign of localization between nucleoids, where Z ring proteins would be expected to assemble (Fig. 3, panels B and C respectively). In the FtsZ depletion experiment, we also stained for PBP 2B and its localization was also dispersed, consistent with expectation that its divisome localization depends on FtsZ. These results are consistent with PBP 3 being mainly associated with the cell division machinery and dependent on the presence of FtsZ.

### PBP 3 shows significant similarity to PBP 2A of *S. aureus*

PBP 3 has the sequence motifs characteristic of a class B PBP (Murray *et al*., 1996). Sequence comparisons revealed that PBP 3 is strongly conserved in the Bacilli, with many strains encoding a protein with substantial similarity (> 48% identity). Interestingly, PBP 3 exhibits a similar degree of relatedness (41% identity for the entire gene) to the *S. aureus* PBP 2A (*Sa*PBP 2A), which is encoded by *mecA* (Lim and Strynadka, 2002), including presence of the domain MecA‐N (pfam: 05223) which is infrequently present in PBPs (Supporting Information Fig. S4). *Sa*PBP 2A is an accessory PBP that endows resistance to β‐lactam antibiotics, being largely responsible for the MRSA phenotype (Hartman and Tomasz, 1984). The suggestion that PBP 3 might have a related function to both PBP 2B and *Sa*PBP 2A prompted us to conduct a series of experiments to determine the role of PBP 3 in resistance to β‐lactam antibiotics.

### Disruption of *pbpC* increases sensitivity to specific β‐lactams

**Figure 4:**
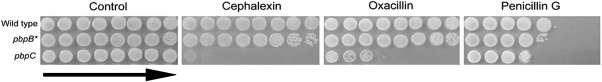
Resistance to specific types of β‐lactam antibiotics is a function of PBP 3. An exponentially growing culture of each bacterial strain was diluted to a specific optical density and used as the starting point for a fourfold dilution series. Samples (10 μl) of the dilutions were spotted onto nutrient agar plates containing the antibiotics Cephalexin (A; 0.08 μg ml^−1^), Oxacillin (B; 0.04 μg ml^−1^), Penicillin G (C; 0.005 μg ml^−1^) and no antibiotic (D). Plates were then incubated at 37°C for 18 h prior to being photographed.

Antibiotic sensitivity tests showed that the loss of PBP 3 made cells more sensitive to certain β‐lactams compared to the wt strain (Fig. 4). The strain carrying the Δ*pbpC* mutation (4015) only grew on plates containing oxacillin and cephalexin when inoculated at significantly higher densities (∼1000 fold) than the wild type or the other *pbp* null mutants tested (*ponA*, strain 4014; *pbpD*, 4013; *pbpE*, 4011 and *pbpF*, 4012) except a strain lacking PBP 2A. The latter strain was more sensitive to oxacillin and cephalexin, which could be related to the mild growth defect that this null mutation causes (Murray *et al*., 1998). However, the reasons behind the increased sensitivity of the *pbpC* mutant were less clear. Microtitre based MIC tests (Table 2) also demonstrated a clear increase in sensitivity to both oxacillin and cephalexin for a strain deleted for *pbpC*, whereas there was no significant change in the MIC for penicillin G.

**Table 2:** MICs of specific β‐lactams.

	MIC (µg ml^−1^)^a^			
β‐lactam Strain	Oxacillin	Cephalexin	Penicillin G	Cefoxitin
168	0.028	0.6	2.4	0.03
(wildtype)	(0.032–0.016)	(1.0–0.5)	(4–0.8)	(0.04–0.01)
4001	0.03	0.8	2.8	0.004
(*pbpB* ^*^)	(0.032–0.016)	(1.0–0.5)	(4–0.8)	(0.005–0.003)
4015	0.008	0.16	2.6	0.02
(*pbpC*)	(0.01–0.005)	(0.2–0.05)	(4–0.4)	(0.04–0.01)

**a.** Value in brackets indicate the maximum and minimum values obtained for MICs for individual experiments.

The crystal structure of *Sa*PBP 2A with several β‐lactams suggested that the reason behind the resistance of *Sa*PBP 2A to certain β‐lactams is the structure of the TPase active site of the protein (Otero *et al*., 2013). Such observation suggests that *Sa*PBP 2A provide the TPase activity required to crosslink the peptides in PG when other *Sa*PBPs are blocked by β‐lactams. To test if PBP 3 in *B. subtilis* has a comparable resistance mechanism to β‐lactams, several mutants including mutants with inactive PBP 2B^(S309A)^ or PBP 3 deleted were tested against oxacillin. As shown in Fig. 4, cells lacking PBP 3 showed increased sensitivity to oxacillin compared to wild‐type cells. To further investigate the resistance mechanism of PBP 3 to oxacillin, either a wild type copy of the *pbpC* gene or a mutant copy of *pbpC* (pbpC*) analogous to the pbpB* (S309A) mutation, were introduced in the *amyE* locus under the control of P_*hyper‐spank*_ promoter. In the absence of IPTG, all mutants lacking PBP 3 showed increased sensitivity to oxacillin compared to wild type (Supporting Information Fig. S3, panel A). The addition of IPTG, which allowed the expression of one of the *pbpC* alleles, *pbpC* or pbpC*, showed that the ectopic expression of PBP 3 but not PBP 3^(S410A)^ decreased the sensitivity of cells to oxacillin, restoring it to almost wild type levels. These results confirm that PBP 3 is the ‘resistance allele’ and that the active site serine was required for β‐lactam resistance. Previous analyses have indicated that both oxacillin and cephalexin may have specificity towards PBPs involved in cell division in *B. subtilis*, which suggests that PBP 3 could potentially be acting redundantly to PBP 2B that is more sensitive to certain β‐lactams (Stokes *et al*., 2005). It was also found that ectopic expression of PBP 3* as a competitor for the natively expressed PBP 3 had no significant effect on cell viability, cell morphology or β‐lactam resistance (Supporting Information Fig. S3, panel A).

Previous transcriptional analysis by Nicolas *et al*. had indicated that *pbpC* was constitutively expressed and not subject to upregulation by stress. However, it has been shown that antibiotic exposure leads to the induction of a diverse set of genes under the control of various ECF sigma factors, particularly SigM, and this increased expression permits the cell to grow in the presence of antibiotics. To determine if the PBP 3 had a role in this ‘resistance’ mechanism, we analysed the sensitivity of a strains lacking both *pbpC* and *sigM* to oxacillin, penicillin G and moenomycin (as a non β‐lactam cell wall synthesis inhibitor) (Supporting Information Fig. S5) compared to isogenic single null mutants and a strain lacking all 7 ECF sigma actors (BSU2007). Loss of PBP 3 had no effect on moenomycin resistance, whereas a strain lacking *sigM* was significantly more sensitive (as was a strain lacking multiple ECF genes (BSU2007); as seen by Lou and Helmann, 2012). Thus, PBP 3 probably has no role in the ECF mediated resistance to cell wall inhibitors. However, when penicillin G and oxacillin were used the effects of the *sigM* and *pbpC* mutations were additive, with *pbpC* apparently contributing more to the sensitivity (Supporting Information Fig. S5).

### β‐lactam binding specificity of PBPs

The increased sensitivity of the *pbpC* mutant was consistent with the notion that PBP 3 has a protective role against the action of β‐lactam antibiotics. If so, this should be detectable at the biochemical level via differences in the specificity of oxacillin and cephalexin binding to the PBPs expressed in *B. subtilis*.

**Figure 5:**
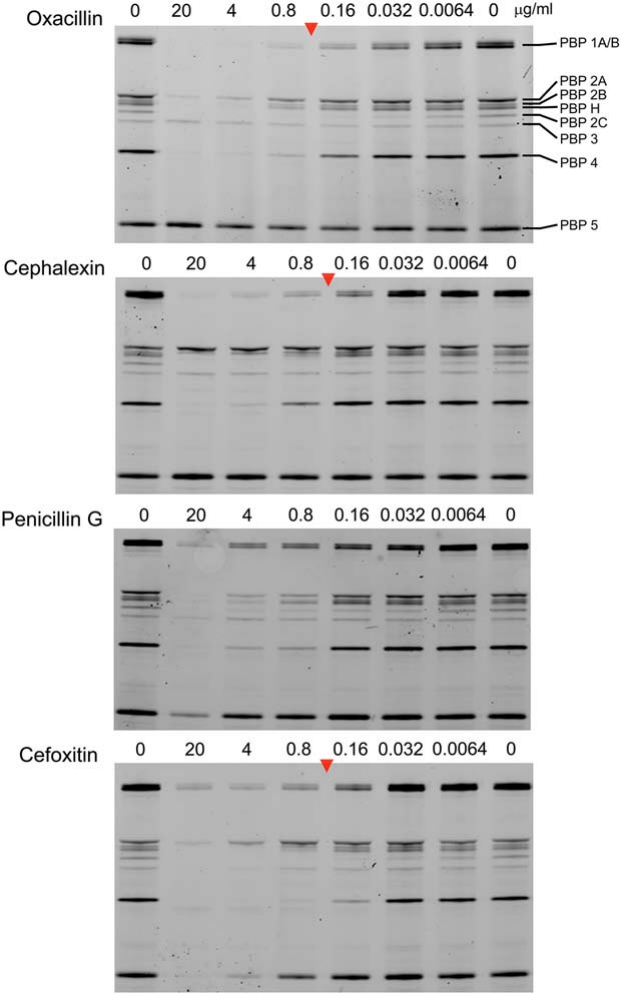
PBP profiles from *in vivo* labelled bacterial strains. Image obtained by fluorography showing the binding profile of bocillin–FL to *B. subtilis* PBPs after separation by SDS‐PAGE. The identity of each PBP is shown on the right of the panel and the labels along the top of each gel indicates the pre‐treatment of the culture prior to being incubated with 0.5 μg ml^−1^ bocillin‐FL and total protein being extracted and separated by SDS‐PAGE.

To test this, we developed an assay based on direct binding of bocillin‐FL (Gutmann *et al*., 1981; Zhao *et al*., 1999) to live cells, bypassing the need to purify membranes and allowing rapid and reproducible processing of samples (Fig. 5). (Note that in these experiments PBP 2B overlaps with PBP H, rather than PBP 2A, for reasons that are not clear.) The bocillin‐FL labelling profiles of wild type culture samples pre‐treated with β‐lactams revealed that one higher molecular weight PBP and PBP 5 (Atrih *et al*., 1999) (asterisks in Fig. 5) were relatively refractory to binding of any of the three compounds at the concentrations tested. The former protein was identified as PBP 3 by absence of the corresponding fluorescent band in the PBP profile of a *pbpC* null mutant (Supporting Information Fig S3; panel B, lane Δ*pbpC* compared to the WT). To exclude the possibility that the poor binding of oxacillin and cephalexin to PBP 3 was due to inability of these compounds to access the active site *in vivo*, a range of other β‐lactams were screened for their ability to bind PBP 3. From this analysis, it was clear that although oxacillin and cephalexin did not show strong affinity for PBP 3, cefoxitin (Supporting Information Fig. S5) did exhibit binding to PBP 3 at comparable concentrations to other β‐lactams under the same conditions, showing that prior treatment with at least one β‐lactam can prevent bocillin‐FL binding to PBP 3. It was also found that a strain lacking active PBP 2B (pbpB*; 4001) exhibited increased sensitivity to cefoxitin (Table 2). It was also notable that the strains lacking functional PBP 2B were more filamentous when cultured in media containing sub‐inhibitory concentrations of cefoxitin compared to the wild‐type strain.

## Discussion

The results presented here show that PBP 3 is important for β‐lactam resistance in *B. subtilis* and that it has a crucial role in enabling cell division when the catalytic activity of PBP 2B is compromised. As such, PBP 3 may provide a fail‐safe mechanism that can rescue cells from the potentially catastrophic division failure. The inactivation of PBP 2B homologue in *S. pneumoniae*, PBP 2X, was lethal (Peters *et al*., 2014), probably due to the absence of any other PBP that could functionally supply the transpeptidase activity necessary for cell division. Many relatives of *B. subtilis*, have recognisable PBP 3 homologues, suggesting that this back up to the division‐associated TPase is a common function in this bacterial family. Interestingly, database searches excluding the *Bacilli* revealed PBP 3 to have similarity to *Sa*PBP 2A in methicillin‐resistant *Staphylococcus aureus* (MRSA). *Sa*PBP 2A is responsible for β‐lactam tolerance in MRSA and it works by providing a TPase with a low affinity for β‐lactam antibiotics that is able to function in cell wall synthesis when the other PBPs are inhibited. As for PBP 3 in *B. subtilis*, *Sa*PBP 2a endows *S. aureus* with resistance to a range of β‐lactams, not least high level resistance to oxacillin, which is a distinguishing marker for MRSA. As PBP 3 homologues are present in a wide range of *Bacillus* spp. few if any of which are pathogenic, it is unlikely to have been acquired as a result of man's use of antibiotics. Our results therefore lend support to the idea that β‐lactam antibiotics have exerted selective evolutionary pressure long before they were exploited by man. In accordance with previous speculation (Kreiswirth *et al*., 1993), our observations on the role of PBP 3 in *Bacillus* suggest that a mechanism to protect the cell from an abortive attempt to divide may be either very ancient in origin or a result of convergent evolution resulting in a similar solution to abortive division in the Entero/Staphylococci through clinical use of β‐lactams. In this respect, the data presented here has strong parallels with that seen for PBP 2A in *S. aureus* (Pinho and Errington, 2005). However, the constitutive expression of PBP 3 during vegetative growth in addition to the active recruitment of PBP 3 to the assembling divisome suggests that PBP 3 might have an active role in septal PG synthesis and does not only function as a backup under antibiotic stress (Murray *et al*., 1996; Nicolas *et al*., 2012).

The results presented here provide a functional role for another PBP encoded in the genome of *B. subtilis* and expressed in vegetative growth. Using this data and previous analyses (Popham and Setlow, 1996; Kawai *et al*., 2009, 2011), it is apparent that there is significant functional redundancy for these cell wall synthetic enzymes. This highlights the functional importance of correct wall synthesis for bacterial viability and the ability to adapt to rapidly changing growth conditions or exposure to inhibitory compounds. PBP 3 does not appear to be upregulated upon cell wall stress, despite the existence of a diverse set of transcriptional regulation systems designed to respond to cell wall perturbation [e.g., ECF signal factors (Helmann, 2002)], but is constitutively expressed (see Supporting Information data). Specifically, the ECF sigma factor SigM is thought to play an important role in intrinsic resistance to antibiotics in *B. subtilis* (Lou and Helmann, 2012). However, a strain with *sigM* and *pbpC* mutations was more sensitive against β‐lactams than the single mutants suggesting an additive effect. These results support the premise that PBP 3 contributes to the intrinsic resistance of *B. subtilis* to β‐lactams independently of SigM (Supporting Information Fig. S5). These results could be interpreted as indicating that the process of septum formation during cell division is prone to perturbations that result in the need for functionally redundant enzymes that provide a robust system to avoid abortive cell division.

In the light of these results, we seem to have identified a point at which the septal PG synthesis can be arrested where it seems that full assembly of the complex has occurred. This is consistent with the results obtained by Bisson‐Filho *et al*. (2017) looking at the dynamics of the division complex in living cells. Both these results and ours suggest that the lack of the key biochemical activity provided by PBP 2B or PBP 3 can block the constriction of the division site. Consequently, we are now focused on determining the biochemical role of PBP 2B in the division process and how PBP 3 is able to provide this activity. This potentially explains the delayed division phenotype observed for strains lacking active PBP 2B and offers a novel route toward looking at the dynamics of the division process by microscopy. Following submission of this work, similar results have been obtained by Angeles *et al*. (2017).

## Experimental procedures

### General methods

The strains, plasmids and oligonucleotides used in this study are listed in Table 3.

**Table 3:** Bacterial strains, plasmids and oligonucleotides.

Strain/plasmid	Relevant genotype/features	Source/construction
***B. subtilis***		
168	*trpC2*	Laboratory stock
1801	*trpC2 chr:: pJSIZDpble* (P_*spac*_‐*ftsZ ble*)	(Marston *et al*., 1998)
3941	*trpC2 pbpB^*1–1104*^* ^b^ *lacI aph‐*A3 P*_spac_ pbpB*	pSG5601→168
3105	*trpC2 pbpC^*1–768*^ cat* P*_xyl_ gfp‐pbpC*	(Scheffers *et al*., 2004)
4000	*trpC2 pbpB^*1–1104*^lacI aph‐*A3 P*_spac_ pbpB* *^a^	pSG5662→168
4001	*trpC2 pbpB* ^(^ *^*S309A*^* ^)^	This work
4002	*trpC2 amyE Ω* (*spc* P*_xyl_ gfp‐pbpB*)	pSG5663→168
4003	*trpC2 amyE Ω* (*spc* P*_xyl_ gfp‐pbpB* ^*^)	pSG5664→168
4004	*trpC2 lacI aph‐*A3 P*_spac_ pbpB amyE Ω* (*spc* P*_xyl_ gfp‐pbpB* ^*^)	This work
4005	*trpC2 pbpB* ^*^ *pbpC^*1–768*^ cat* P*_xyl_ gfp‐pbpC*	3105→4001
4006	*trpC2 pbpB* ^*^ *amyE Ω* (*spc* P*_xyl_ gfp‐pbpB*)	4002→4001
4007	*trpC2 pbpC aph‐*A3 *pbpC^*739–2004*^*	pSG5665→168
4008	*trpC2 pbpC* ^*^ *aph‐*A3 *pbpC^*739–2004*^*	pSG5666→168
4009	*trpC2 pbpC* ^*^ *pbpB* ^*^ *amyE Ω* (*spc* P*_xyl_ gfp‐pbpB*)	4008→ 4006
PS1805′	*trpC2 ΔpbpE*::*erm* (moved into168CA)	(Popham and Setlow, 1993)
PS1869′	*trpC2 ΔpbpF*::*erm*	(Popham and Setlow, 1993)
PS2022′	*trpC2 ΔpbpD*::*erm*	(Popham and Setlow, 1994)
PS2062′	*trpC2 ΔponA*::*spc*	(Popham and Setlow, 1995)
PS2352′	*trpC2 pbpC*::*cat*	(Murray *et al*., 1996)
PS2465′	*trpC2 pbpA*::(pTMM4) *cat*	(Murray *et al*., 1997)
HB0031′	*trpC2 ΔsigM::kan*	(Luo and Helmann, 2012)
BSU2007′	*168 sigMWXYZV ylaC (Δ7ECF)*	(Asai *et al*., 2008)
DPVB133′	*trpC2 ΔpbpH*::*spc*	(Wei *et al*., 2003)
4011	*trpC2 ΔpbpE*::*erm*	PS1805→168
4012	*trpC2 ΔpbpF*::*erm*	PS1869→168
4013	*trpC2 ΔpbpD*::*erm*	PS2022→168
4014	*trpC2 ΔponA*::*spc*	PS2062→168
4015	*trpC2 pbpC*::*cat*	PS2352→168
4016	*trpC2 pbpA*::(pTMM4) *cat*	PS2465→168
4017	*trpC2 ΔpbpH*::*spc*	DPVB133→168
4018	*trpC2 pbpB* ^*^ *ΔpbpE*::*erm*	4011→ 4001
4019	*trpC2 pbpB* ^*^ *ΔpbpF*::*erm*	4012→ 4001
4020	*trpC2 pbpB* ^*^ *ΔpbpD*::*erm*	4013→ 4001
4021	*trpC2 pbpB* ^*^ *ΔponA*::*spc*	4014→ 4001
4022	*trpC2 pbpB* ^*^ *pbpC*::*cat*	4015→ 4001
4023	*trpC2 pbpB* ^*^ *pbpA*::(pTMM4) *cat*	4016→ 4001
4024	*trpC2 pbpB* ^*^ *ΔpbpH*::*spc*	4017→ 4001
KS50	*trpC2 amyE Ω (spc* P*_spank_ pbpC)*	pKS4→168
KS51	*trpC2 pbpB* ^*^ *amyE Ω (spc* P*_spank_ pbpC)*	pKS4→4001
KS52	*trpC2 amyE Ω (spc* P*_spank_ pbpC) pbpC::cat*	4015→KS50
KS53	*trpC2 amyE Ω (spc* P*_spank_ pbpC* ^*^ *)*	pKS5→168
KS54	*trpC2 pbpB* ^*^ *amyE Ω (spc* P*_spank_ pbpC* ^*^ *)*	pKS5→4001
KS55	*trpC2 pbpC::cat amyE Ω (spc* P*_spank_ pbpC* ^*^ *)*	pKS5→4015
RD300	*trpC2 ΔsigM::kan*	HB0031→168
RD301	*trpC2 ΔpbpC::cat ΔsigM::kan*	RD300→4015
***E. coli***		
DH 5α	*F‐endA1 hsdR17 supE44 thi‐1 λ‐recA1 gyrA96 relA1 Δ(lacZYA‐argF) U169 Φ80 δlacZ ΔM15*	Laboratory stock
NM554	*recA13 araD139* Δ(*ara‐leu*)*7696* Δ(*lac*)*l7A galU galK hsdR rpsL* (Str^r^) *mcrA mcrB*	Laboratory stock
**Plasmids**		
pSG441	*bla aph‐*A3 *lacI* P*_spac_*	(Illing and Errington, 1991)
pSG5601	*bla aph‐*A3 *lacI* P*_spac_ pbpB^*1–1104*^*	This work
pSG5662	*bla aph‐*A3 *lacI* P*_spac_ pbpB^*1–1104*^* ^*^	This work
pQE31	*ori* (ColE1) *bla T5 promotor/lac* operator	Qiagen
pSG5670	pQE31 *pbpB*	This work
pSG5671	pQE31 *pbpB* ^*^	This work
pSG1729	*bla amyE3*′ *spc* P*_xyl_ gfp amyE5*′	(Lewis and Marston, 1999)
pSG5663	*bla amyE3*′ *spc* P*_xyl_ gfp‐pbpB amyE5*′	This work
pSG5664	*bla amyE3*′ *spc* P*_xyl_ gfp‐pbpB* ^*^ *amyE5*′	This work
pSG5045	*bla cat P_xyl_ gfp‐pbpC^*1–768*^*	(Scheffers *et al*., 2004)
pUK19	*aph‐*A3 *bla*	Gift from W. G. Haldenwang
pSG5665	*aph‐*A3 *bla pbpC^*739–2004*^*	This work
pSG5666	*aph‐*A3 *bla pbpC^*739–2004*^* ^*^	This work
pDR111	*bla amyE3*′ *spc* P*_spank_ lacI amyE5*′	(Vavrová *et al*., 2010)
pKS4	*bla amyE3*′*spc* P_*spank*_ *pbpC lacI amyE5*′	This work
pKS5	*bla amyE3*′ *spc* P_*spank*_ *Ω pbpC* ^*^ *lacI amyE5*′	This work
**Oligonucleotides** ^d^		
PBPB‐F	GATggatCCAAAAAAGAATAAATTTATGAATAGAGGAGC	
PBPB‐R	ACTggtaccTTAATCAGGATTTTTAAACTTAACCTTG	
SDM B‐F	GAACCCGGGGCCACGATGAAGATCTTTACACTCGC	
SDM B‐R	CGTGGCCCCGGGTTCATACGCATATGAAATCAAATC	
pbpB‐1	GCAtctagaAGCGCATTATGGACATTG	
pbpB‐2	CAGTTTgcatgcAGCGACATTCGACGACCTTAG	
pbpB‐3	ATCggatccCCAAAAAAGAATAAATTTATGAATAGAGGAGCAGC	
pbpB‐4	ATCgtcgacTTTAATCAGGATTTTTAAACTTAACCTTGATTACGG	
yllA‐F	AGAATTCAAAATAGCATTAAGCCGCTTCTTGCG	
ftsL‐R	GCATTTGAATCATTCCTGTATGTTTTTCACTTTTTTATCTTTTAAATTCAAGCCG	
pbpC‐1	ATCggatccTCAGCTTCAAGAATACTGCTGTGCTG	
pbpC‐2	ATGctgcagTTTAATTCGATTGAAATTGCTTTTTCGCTTTCTC	
SDM C‐F	GACATACGCGCCAGGTGCTACCATTAAACCGATTGCGGC	
SDM C‐R	GGTTTAATGGTAGCACCTGGCGCGTATGTCTTATTGAAT	
pbpC‐3	GATgcatgcGAGGGGAAAGTCATGTTAAAAAAGTGTATTCTACTAG	
pbpC‐4	ATCgcatgcGCCCCCTTACTAGTTCATTCGGCCTCAGATCC	

**a.** Numbers indicate the base coordinates of the gene cloned from http://genolist.pasteur.fr/SubtiList/.

**b.** Numerical values indicate the region of coding sequence present (starting from the initiation codon).

**a** ^*^indicates that the active site of the PBP was mutated, in the case of *pbpB* this corresponds to S309A, where the codon corresponding to residue S 309 of protein was mutated to A. For *pbpC*, serine 410 was mutated to alanine.

**d.** Lower case letters show where restriction sites were introduced into the oligonucleotide sequence.

*B. subtilis* strains were transformed according the method of (Anagnostopoulos and Spizizen, 1961) as modified by (Jenkinson, 1983). Simple genetic constructions where markers were moved from one background to another are described in Table 3, whereas more complex strain constructions are described below.

DNA manipulations and *E. coli* transformations were carried out using standard methods (Sambrook *et al*., 1989). Plasmid DNA was purified using DNA purification systems of Qiagen and Promega according to the manufacturer's instructions.

*B. subtilis* was cultured on nutrient agar (Oxoid) as a solid medium, and antibiotic medium 3 (Difco) for liquid cultures. Genetic constructs were selected for using kanamycin at 5 µg ml^−1^, chloramphenicol at 5 µg ml^−1^ and spectinomycin at 50 µg ml^−1^. IPTG (0.5 mM) and/or xylose (0.5%) were added as necessary. *E. coli* strains were cultured on nutrient agar or in 2YT (Sambrook *et al*., 1989), supplemented with ampicillin (100 µg ml^−1^) as required.

Protein samples from *B. subtilis* and Western blotting was done as described by Daniel *et al*. (2000). Strain constructions were confirmed both by antibiotic resistance, dependence upon specific inducers (where appropriate) and by the use of PCR amplification across the regions of insertion, combined with DNA sequencing of the region if required.

### Determination of sensitivity to β‐lactam antibiotics

Serial dilutions of test β‐lactams in PAB and inoculated with between 100 and 5000 cells in a final volume of 200 μl (confirmed post analysis by conventional CFU determination on nutrient agar plates) and incubated at 37°C in a BMG microtitre plate reader with shaking. The optical density of the cultures was then measured after 8 h incubation at 37°C. The lowest concentration of antibiotic preventing growth of the culture was defined as the MIC. The results of 8 independent assay are summarised in Table 2. However, as this represented a very small bacterial culture, it was impractical to use for analytical methods so the concentration of antibiotic necessary to lyse a culture at an optical density (600 nm) of 0.2 was determined and denoted the Minimum Lytic Concentration (MLC). The MLC values for of oxacillin, cephalexin and penicillin G for 168CA grown in PAB were determined to be 0.3 (± 0.1), 0.8 (± 0.15) and approximately 20 (± 4) μg ml^−1^ respectively.

To allow comparative sensitivity of strains, cultures of the test strains were incubated overnight at 30°C in PAB medium and then diluted 1:10 in the same medium and incubated for a further 1 h at 37°C. After this time, the cultures were diluted to OD_600_ = 1.0 and a series of fourfold dilutions were made using SMM. A 10μl spot of each dilution serial was dropped onto nutrient agar (NA) plates containing cephalexin, oxacillin or penicillin G as well as onto a NA plate with no antibiotic. The plates were then incubated at 37°C for 24 h before being photographed. The antibiotic concentrations required to detect differential strain sensitivity were determined empirically (using the MIC values as a guide). Form this analysis, it was found that 0.08 µg ml^−1^ cephalexin, 0.04 µg ml^−1^ oxacillin and 0.005 µg ml^−1^ penicillin G gave the best differentiation and reproducible results.

### Elimination of the transpeptidase activity of PBP 2B by the S309A mutation

To test the ability of PBP 2B and PBP 2B^(S309A)^ to bind penicillin, the coding sequence of *pbpB* was amplified by PCR from genomic DNA of strain 168 (using oligonucleotide primers PBPB‐ F and R), digested with *Bam*HI and *Kpn*I and ligated with similarly digested pQE31. The transformation of this DNA into NM554 (pREP4) allowed the isolation of pSG5670 in which the wild‐type *pbpB* gene was expressed from the IPTG‐inducible promoter of pQE31. The S309A mutation was then introduced into pSG5670 by site directed mutagenesis (primers SDM B‐F and R) to give pSG5671.

Cultures of NM554 (pREP4) with pSG5670, 5671 or NM554 alone (as a negative control) were grown in 2YT to an optical density (600nm) of 0.5, at which point IPTG was added to the cultures and incubation continued for 1 h at 37°C. Samples (50ml) of each culture were then harvested and the cell pellets were washed by PBS and then suspended in 15 ml PBS and held on ice. The cells in each sample were broken by three passages through a French Press (2,000 lb in^−2^). The resulting cell lysates were centrifuged at 2,000 r.p.m. in a bench top centrifuge for 20 min to remove unbroken cells and large bits of debris. The supernatants were then centrifuged at 80,000 r.p.m. for 30 min at 4°C to pellet membrane vesicles. The resulting pellets were suspended in 400 μl PBS by sonication for 2 s. The resulting suspension was then divided into aliquots of 100 μl and stored at −20°C.

The affinity of the PBP 2B and PBP 2B^(S309A)^ for penicillin was determined by adding 2 μl (1 mg ml^−1^) of bocillin‐FL (Invitrogen) to 100 µl of the cell membrane samples, followed by incubation at room temperature for 15 min. 100 µl 2 × SDS was then added to each sample and the proteins denatured at 99°C for 2 min prior to being separated by SDS‐PAGE. The proteins with covalently bound bocillin‐FL were then identified by scanning the protein gel for fluorescent emission using a Fuji FLA3000 scanner (Zhao *et al*., 1999). The protein gel was stained with Coomassie Blue (R250; Sigma) to confirm that approximately equal amounts of *E. coli* total membrane proteins and PBP 2B and PBP 2B^(S309A)^ had been loaded (Fig. 1). A fluorogram of the gel (Fig. 1) showed that only the wild type PBP 2B was fluorescent, with no detectable fluorescence in the equivalent position in the lane with the mutant protein, although Coomassie staining showed that similar amounts of protein had been loaded, confirming that the covalent binding of penicillin to PBP 2B was abolished by the S309A substitution.

### Construction of strains with conditional expression of modified *pbpB* genes

To allow repressible expression of *pbpB* and to introduce mutations into the genome of *B. subtilis*, plasmid pSG5601 was constructed such that the 5′ portion of the *pbpB* gene, comprising the RBS and the coding sequence of the first 368 amino acids, using PCR primers pbpB‐1 and pbpB‐2 to amplify the gene and cloning it into pSG441 such that the RBS of *pbpB* gene was placed in front of the *P_spac_* promoter. Plasmids pSG5601 was then transformed into 168 selecting for the kanamycin resistance in the presence of IPTG. A clone that was IPTG dependent for growth for isolated and designated strain 3941.

To determine the functionality of PBP 2B^(S309A)^, the wild‐type *pbpB* was amplified by PCR, with primers pbpB‐3 and ‐4, and the resulting DNA fragment inserted into plasmid pSG1729 to give plasmid pSG5663; this construct was designed to produce a GFP fusion to PBP 2B that was under the control of the P_*xyl*_ promoter and could be integrated into the *amyE* locus of *B. subtilis*. Site directed mutagenesis was then used to change the Serine 309 codon of *pbpB* to encode alanine (using primers SDM B‐F and R), resulting in plasmid pSG5664. Plasmids pSG5663 and pSG5664 were transformed into strain 168 to give strains 4002 and 4003 respectively, giving strains with a second copy of either wild type or mutant PBP 2B (PBP 2B^(S309A)^) under the control of the P_*xyl*_ promoter. To determine if the inducible PBP 2B^(S309A)^ genes could complement depletion of the wild protein, the P_*spac*_ promoter was inserted immediately upstream of *pbpB* by transforming strain 4003 with a DNA fragment amplified by PCR, spanning from *yllA* to the end of *ftsL* (primers used yllA‐F and ftsL‐R), ligated to pSG5601, digested with *Sph*I and blunted by Klenow. Selection for kanamycin resistance in the presence of IPTG gave rise to several transformant colonies that were then screened for kanamycin and spectinomycin resistance as well as dependence on IPTG for cell growth. A single clone (strain 4004) was then taken and the location of the insertion of the P_*spac*_ promoter, *lacI* and the kanamycin resistance cassette between the stop codon of *ftsL* and the RBS of *pbpB* confirmed by PCR and sequencing.

As it was found that strain 4004 was viable when grown in the presence of xylose alone, indicating that PBP 2B^(S309A)^ may function for cell division, site directed mutagenesis (primers SDM B‐F and‐R) was then used to introduce the S309A mutation into pSG5601, creating plasmid pSG5662. Plasmid pSG5662 was then integrated into 168 and the location of the pbpB* (S309A) mutation determined (either in the truncated copy of *pbpB* under the native promoter or in the full length copy under the P_*spac*_ promoter). A clone of 168 with pSG5662 integrated into the chromosome was then isolated, where the S309A mutation was located in the functional copy of *pbpB*, as determined by PCR and sequencing, and denoted strain 4000. Strain 4000 was then grown in the absence of IPTG to promote the excision of the integrated plasmid. An isolate resulting from this technique, strain 4001, was found to have the pbpB* mutation without any of the plasmid sequences used to introduce the mutation.

To determine the phenotype of a strain with catalytically inactive PBP 2B and PBP 3, strain 4009 was constructed in 3 steps. Firstly, chromosomal DNA from strain 4002 (P*_xyl_* inducible *gfp*‐*pbpB*) was transformed into strain 4001 (with pbpB*) to give strain 4006 containing both pbpB* at the chromosomal locus and a P*_xyl_* inducible wild‐type copy of *pbpB* fused to GFP at *amyE*. Secondly, the C‐terminal part of *pbpC* was amplified by PCR (primers pbpC‐1 and −2) inserted into plasmid pUK19, to give plasmid pSG5665. Site directed mutagenesis of pSG5665 using oligonucelotides SDM C‐F and –R was then used to change the codon for the catalytic serine at position 410 to encode for alanine, resulting in plasmid pSG5666. Finally, plasmid pSG5666 was transformed into strain 4006 selecting for kanamycin resistance in the presence of xylose. The resulting transformants were then screened for xylose dependence and then PCR used to confirm that the genotype of the strain was as expected, resulting in strain 4009.

### Construction of conditional alleles of *pbpC*

To increase the cellular abundance of PBP 3, we cloned the *pbpC* gene including the native ribosome binding site (PCR amplified using oligos pbpC‐3 and −4) was cloned into pDR111 digested with *Sph*I to give pKS4. This plasmid was then used as a template for site directed mutagenesis to produced pKS5 where the active site serine of PBP 3 was replaced by alanine (S410A) as described above. These plasmids were then used to generate strains KS50 and KS53 by transformation and screening for loss of amylase activity to confirm insertion of the IPTG inducible copies of *pbpC* into the *amyE* locus. Strains KS51 and KS54 were then constructed by the transformation of strain 4001 with pKS4 and pKS5 respectively. Then finally, strain KS52 was constructed by the introduction of the *pbpC* null mutation from strains 4015 into KS50

### Depletion of PBP 2B

Where lethal effects were predicted or identified, strains were constructed with a complementing copy of the *pbpB* gene under the control of an inducible promoter. To analyse the phenotype of such strains, the culture conditions were manipulated to remove one or both inducers as described in (Daniel *et al*., 2000), and samples taken every 30 min for the measuring of OD_600_, microscopy and Western blot analysis where required.

### Cellular abundance of PBP 2B and GFP‐PBP 2B^(S309A)^

To ensure that the normal growth of strain 4004 was not due to residual expression of the wild type gene, the total protein content of strain 4004 grown under the conditions described above was analysed by Western blotting using antiserum specific for PBP 2B (Fig. 1C). The results showed that a small amount of wild‐type PBP 2B was present in the culture grown in the absence of IPTG after incubation for 1.0 h, but cell division was clearly perturbed in cultures lacking both xylose and IPTG by this time, and by 1.5 h cell lysis was evident.

### Microscopy

For microscopy, cells from exponentially growing cultures were mounted on a thin film of 1% agarose in SMM medium (Anagnostopoulos and Spizizen, 1961), essentially as described previously (Glaser *et al*., 1997). To stain cell membranes, Nile red or FM5.94 was added to a sample of the culture to a final concentration of 2µg ml^−1^ prior to mounting on an agarose slide.

For the subcellular localisation by immunofluorescence of PBP 3 and PBP 2B, the strains were grown to mid exponential phase of growth (OD_600_ ∼ 0.5). For the localisation of PBP 2B and PBP 3 in cells depleted of PBP 2B or FtsZ, the strains 3941 and 1801 were used, respectively, following the depletion protocol described in (Daniel *et al*., 2000). Subsequently, samples were fixed by the addition of an equal volume of ice cold fixation buffer (5% paraformaldehyde in PBS) and held on ice for at least 30 min. Cells were washed 3× in PBS and suspended in GTE buffer (50 mM Glucose, 25 mM Tris/HCl pH 8 and 10 mM EDTA pH 8). The cell suspension was then spotted on a dry multiwell slide and allowed to stand for 5 min. The solution was aspirated off and the slide left to dry. Poly‐lysine (0.01%) was spotted onto the cells and left for 2 min, aspirated off and allowed to air dry. Cell spots were then treated with lysozyme (10 mg ml^−1^ in GTE) ∼2 min, washed with PBS and allowed to dry. Cells were re‐hydrated with PBS for 2 min then blocked with PBS/2% BSA for 15 min. Primary antibody was added to cells and incubated overnight at 4°C. The cell spots were then washed 10 × with PBS before applying the secondary antibody (1/10,000 dilution in PBS/2% BSA) to the slide and incubating at room temperature in the dark for 1.30 h. Spots were then washed 10× with PBS, DAPI (0.2 µg ml^−1^ in antifade (ProLong Gold; Invitrogen) was used as a mountant and a coverslip applied.

Microscopic images were taken using Nikon TiE microscope coupled to a Hamamatsu C9100 EMCCD or a Sony CoolSnap HQ2 camera operated by Metamorph 7 imaging software (Universal imaging). All images were analysed with Metamorph 7 imaging software. Python27 software was used to sort the fluorescence data and ImageJ software was used to create the heat maps (Fig. 2), whereas Adobe Photoshop version 7.0.1 was used to construct figures.

### PBP profile determination and β‐lactam specificity

To provide sufficient protein for the analysis of the PBP profiles, cultures were grown to an OD_600_ of 0.2 in PAB (with IPTG (1 mM where required) and then 0.5 μg ml^−1^ bocillin‐FL added directly to the culture medium and incubation for 1 min at RT to allow binding of the penicillin. The cells were then harvested and broken by sonication for 15 s on ice. Total protein extracts were resolved by SDS‐PAGE and the resulting gels scanned using a Typhoon scanner (GE healthcare).

To determine the specificity of other β‐lactams compared to bocillin‐FL, the cultures were pre‐treated with the β‐lactam (oxacillin, cephalexin, ceftriaxone or penicillin G) for 2 min prior to the addition of bocillin‐FL. The cells were then treated as described above to allow the PBP profile to be identified.

## Supporting information

Supporting Figures

## References

[mmi13765-bib-0001] Anagnostopoulos, C. , and Spizizen, J. (1961) Requirements for transformation in *Bacillus subtilis* . J Bacteriol 81: 741–746. 16561900 10.1128/jb.81.5.741-746.1961 PMC279084

[mmi13765-bib-0002] Asai, K. , Ishiwata, K. , Matsuzaki, K. , and Sadaie, Y. (2008) A viable *Bacillus subtilis* strain without functional extracytoplasmic function sigma genes. J Bacteriol 190: 2633–2636. 18223082 10.1128/JB.01859-07 PMC2293210

[mmi13765-bib-0003] Atrih, A. , Bacher, G. , Allmaier, G. , Williamson, M.P. , and Foster, S.J. (1999) Analysis of peptidoglycan structure from vegetative cells of *Bacillus subtilis* 168 and role of PBP 5 in peptidoglycan maturation. J Bacteriol 181: 3956–3966. 10383963 10.1128/jb.181.13.3956-3966.1999 PMC93885

[mmi13765-bib-0101] Bi, E.F. , and Lutkenhaus, J. (1991) FtsZ ring structure associated with division in *Escherichia coli* . Nature 14: 161–164. 10.1038/354161a0 1944597

[mmi13765-bib-0004] Bisson‐Filho, A.W. , Hsu, Y.P. , Squyres, G.R. , Kuru, E. , Wu, F. , Jukes, C. , *et al* (2017) Treadmilling by FtsZ filaments drives peptidoglycan synthesis and bacterial cell division. Science 355: 739–743. 28209898 10.1126/science.aak9973 PMC5485650

[mmi13765-bib-0006] Commichau, F.M. , Dickmanns, A. , Gundlach, J. , Ficner, R. , and Stülke, J. (2015) A jack of all trades: the multiple roles of the unique essential second messenger cyclic di‐AMP. Mol Microbiol 97: 198–204. 10.1111/mmi.13026 25869574

[mmi13765-bib-0007] Daniel, R.A. , Williams, A.M. , and Errington, J. (1996) A complex four‐gene operon containing essential cell division gene *pbpB* in *Bacillus subtilis* . J Bacteriol 178: 2343–2350. 8636036 10.1128/jb.178.8.2343-2350.1996 PMC177943

[mmi13765-bib-0008] Daniel, R.A. , Harry, E.J. , and Errington, J. (2000) Role of penicillin‐binding protein PBP 2B in assembly and functioning of the division machinery of *Bacillus subtilis* . Mol Microbiol 35: 299–311. 10652091 10.1046/j.1365-2958.2000.01724.x

[mmi13765-bib-0009] Daniel, R.A. , Noirot‐Gros, M.F. , Noirot, P. , and Errington, J. (2006) Multiple interactions between the transmembrane division proteins of Bacillus subtilis and the role of FtsL instability in divisome assembly. J Bacteriol 188: 7396–7404. 16936019 10.1128/JB.01031-06 PMC1636283

[mmi13765-bib-0010] Datta, P. , Dasgupta, A. , Singh, A.K. , Mukherjee, P. , Kundu, M. , and Basu, J. (2006) Interaction between FtsW and penicillin‐binding protein 3 (PBP3) directs PBP3 to mid‐cell, controls cell septation and mediates the formation of a trimeric complex involving FtsZ, FtsW and PBP3 in mycobacteria. Mol Microbiol 62: 1655–1673. 17427288 10.1111/j.1365-2958.2006.05491.x

[mmi13765-bib-0011] Glaser, P. , Sharpe, M.E. , Raether, B. , Perego, M. , Ohlsen, K. , and Errington, J. (1997) Dynamic, mitotic‐like behavior of a bacterial protein required for accurate chromosome partitioning. Genes Dev 11: 1160–1168. 9159397 10.1101/gad.11.9.1160

[mmi13765-bib-0012] Goehring, N.W. , and Beckwith, J. (2005) Diverse paths to midcell: assembly of the bacterial cell division machinery. Curr Biol 15: 514–526. 10.1016/j.cub.2005.06.038 16005287

[mmi13765-bib-0013] Goffin, C. , and Ghuysen, J.M. (2002) Biochemistry and comparative genomics of SxxK superfamily acyltransferases offer a clue to the mycobacterial paradox: presence of penicillin‐susceptible target proteins versus lack of efficiency of penicillin as therapeutic agent. Microbiol Mol Biol Rev 66: 702–738. 12456788 10.1128/MMBR.66.4.702-738.2002 PMC134655

[mmi13765-bib-0015] Gutmann, L. , Williamson, R. , and Tomasz, A. (1981) Physiological properties of penicillin‐binding proteins in group A streptococci. Antimicrob Agents Chemother 19: 872–880. 7027926 10.1128/aac.19.5.872 PMC181537

[mmi13765-bib-0016] Hartman, B.J. , and Tomasz, A. (1984) Low‐affinity penicillin‐binding protein associated with beta‐lactam resistance in Staphylococcus aureus. J Bacteriol 158: 513–516. 6563036 10.1128/jb.158.2.513-516.1984 PMC215458

[mmi13765-bib-0017] Helmann, J.D. (2002) The extracytoplasmic function (ECF) sigma factors. Adv Microb Physiol 46: 47–100. 12073657 10.1016/s0065-2911(02)46002-x

[mmi13765-bib-0018] Helmann, J.D. (2006) Deciphering a complex genetic regulatory network: the *Bacillus subtilis* sigmaW protein and intrinsic resistance to antimicrobial compounds. Sci Prog 89: 243–266. 17338440 10.3184/003685006783238290 PMC10368348

[mmi13765-bib-0019] Illing, N. , and Errington, J. (1991) The *spoIIIA* operon of *Bacillus subtilis* defines a new temporal class of mother‐cell‐specific sporulation genes under the control of the s^E^ form of RNA polymerase. Mol Microbiol 5: 1927–1940. 1766372 10.1111/j.1365-2958.1991.tb00816.x

[mmi13765-bib-0020] Jenkinson, H.F. (1983) Alterated arrangement of proteins in the spore coat of a germination mutant of *Bacillus subtilis* . J Gen Microbiol 129: 1945–1958. 6415224 10.1099/00221287-129-6-1945

[mmi13765-bib-0100] Karimova, G. , Dautin, N. , and Ladant, D. (2005) Interaction network among *Escherichia coli* membrane proteins involved in cell division as revealed by bacterial two‐hybrid analysis. J Bacteriol 187: 2233–2243. 15774864 10.1128/JB.187.7.2233-2243.2005 PMC1065216

[mmi13765-bib-0021] Kato, J.‐I. , Nishimura, Y. , Yamada, M. , Suzuki, H. , and Hirota, Y. (1988) Gene organization in the region containing a new gene involved in chromosome partition in *Escherichia coli* . J Bacteriol 170: 3967–3977. 2842295 10.1128/jb.170.9.3967-3977.1988 PMC211397

[mmi13765-bib-0022] Kawai, Y. , Asai, K. , and Errington, J. (2009) Partial functional redundancy of MreB isoforms, MreB, Mbl and MreBH, in cell morphogenesis of *Bacillus subtilis* . Mol Microbiol 73: 719–731. 19659933 10.1111/j.1365-2958.2009.06805.x

[mmi13765-bib-0023] Kawai, Y. , Marles‐Wright, J. , Cleverley, R.M. , Emmins, R. , Ishikawa, S. , Kuwano, M. , *et al* (2011) A widespread family of bacterial cell wall assembly proteins. EMBO J 30: 4931–4941. 21964069 10.1038/emboj.2011.358 PMC3243631

[mmi13765-bib-0024] Kreiswirth, B. , Kornblum, J. , Arbeit, R.D. , Eisner, W. , Maslow, J.N. , McGeer, A. , Low, D.E. , and Novick, R.P. (1993) Evidence for a clonal origin of methicillin resistance in Staphylococcus aureus. Science 259: 227–230. 8093647 10.1126/science.8093647

[mmi13765-bib-0025] Lewis, P.J. , and Marston, A.L. (1999) GFP vectors for controlled expression and dual labelling of protein fusions in *Bacillus subtilis* . Gene 227: 101–109. 9931458 10.1016/s0378-1119(98)00580-0

[mmi13765-bib-0026] Lim, D. , and Strynadka, N.C. (2002) Structural basis for the beta lactam resistance of PBP2a from methicillin‐resistant *Staphylococcus aureus* . Nat Struct Biol 9: 870–876. 12389036 10.1038/nsb858

[mmi13765-bib-0027] Luo, Y. , and Helmann, J.D. (2012) Analysis of the role of *Bacillus subtilis* σ(M) in β‐lactam resistance reveals an essential role for c‐di‐AMP in peptidoglycan homeostasis. Mol Microbiol 83: 623–639. 22211522 10.1111/j.1365-2958.2011.07953.x PMC3306796

[mmi13765-bib-0028] Marston, A.L. , Thomaides, H.B. , Edwards, D.H. , Sharpe, M.E. , and Errington, J. (1998) Polar localization of the MinD protein of *Bacillus subtilis* and its role in selection of the mid‐cell division site. Genes Dev 1: 3419–3430. 10.1101/gad.12.21.3419 PMC317235 9808628

[mmi13765-bib-0029] Massidda, O. , Anderluzzi, D. , Friedli, L. , and Feger, G. (1998) Unconventional organization of the division and cell wall gene cluster of *Streptococcus pneumoniae* . Microbiology 144: 3069–3078. 9846742 10.1099/00221287-144-11-3069

[mmi13765-bib-0031] Morales Angeles, D. , Liu, Y. , Hartman, A.M. , Borisova, M. , de Sousa Borges, A. , de Kok, N. , *et al* (2017) Pentapeptide‐rich peptidoglycan at the Bacillus subtilis cell‐division site. Mol Microbiol 104: 319–333. 28118510 10.1111/mmi.13629

[mmi13765-bib-0032] Murray, T. , Popham, D.L. , and Setlow, P. (1996) Identification and characterization of *pbpC*, the gene encoding *Bacillus subtilis* penicillin‐binding protein 3. J Bacteriol 178: 6001–6005. 8830698 10.1128/jb.178.20.6001-6005.1996 PMC178458

[mmi13765-bib-0033] Murray, T. , Popham, D.L. , and Setlow, P. (1997) Identification and characterization of *pbpA* encoding *Bacillus subtilis* penicillin‐binding protein 2A. J Bacteriol 179: 3021–3029. 9139922 10.1128/jb.179.9.3021-3029.1997 PMC179068

[mmi13765-bib-0034] Murray, T. , Popham, D.L. , Pearson, C.B. , Hand, A.R. , and Setlow, P. (1998) Analysis of outgrowth of *Bacillus subtilis* spores lacking penicillin‐binding protein 2a. J Bacteriol 180: 6493–6502. 9851991 10.1128/jb.180.24.6493-6502.1998 PMC107750

[mmi13765-bib-0035] Nicolas, P. , Mäder, U. , Dervyn, E. , Rochat, T. , Leduc, A. , Pigeonneau, N. , *et al* (2012) Condition‐dependent transcriptome reveals high‐level regulatory architecture in *Bacillus subtilis* . Science 335: 1103–1106. 22383849 10.1126/science.1206848

[mmi13765-bib-0036] Otero, L.H. , Rojas‐Altuve, A. , Llarrull, L.I. , Carrasco‐López, C. , Kumarasiri, M. , Lastochkin, E. , and Hermoso, J.A. (2013) How allosteric control of S*taphylococcus aureus* penicillin binding protein 2a enables methicillin resistance and physiological function. Proc Natl Acad Sci USA 110: 16808–16813. 24085846 10.1073/pnas.1300118110 PMC3800995

[mmi13765-bib-0037] Pares, S. , Mouz, N. , Pétillot, Y. , Hakenbeck, R. , and Dideberg, O. (1996) X‐ray structure of *Streptococcus pneumoniae* PBP2x, a primary penicillin target enzyme. Nat Struct Biol 3: 284–289. 8605631 10.1038/nsb0396-284

[mmi13765-bib-0038] Peters, K. , Schweizer, I. , Beilharz, K. , Stahlmann, C. , Veening, J.W. , Hakenbeck, R. , and Denapaite, D. (2014) *Streptococcus pneumoniae* PBP2x mid‐cell localization requires the C‐terminal PASTA domains and is essential for cell shape maintenance. Mol Microbiol 92: 733–755. 24655324 10.1111/mmi.12588

[mmi13765-bib-0039] Pinho, M.G. , and Errington, J. (2005) Recruitment of penicillin‐binding protein PBP2 to the division site of *Staphylococcus aureus* is dependent on its transpeptidation substrates. Mol Microbiol 55: 799–807. 15661005 10.1111/j.1365-2958.2004.04420.x

[mmi13765-bib-0040] Popham, D.L. , and Setlow, P. (1993) Cloning, nucleotide sequence, and regulation of the *Bacillus subtilis pbpE* operon, which codes for penicillin‐binding protein 4* and an apparent amino acid racemase. J Bacteriol 175: 2917–2925. 8491712 10.1128/jb.175.10.2917-2925.1993 PMC204609

[mmi13765-bib-0041] Popham, D.L. , and Setlow, P. (1994) Cloning, nucleotide sequence, mutagenesis, and mapping of the *Bacillus subtilis pbpD* gene, which codes for penicillin‐binding protein 4. J Bacteriol 176: 7197–7205. 7961491 10.1128/jb.176.23.7197-7205.1994 PMC197107

[mmi13765-bib-0042] Popham, D.L. , and Setlow, P. (1995) Cloning, nucleotide sequence, and mutagenesis of the *Bacillus subtilis ponA* operon, which codes for penicillin‐binding protein (PBP) 1 and a PBP‐related factor. J Bacteriol 177: 326–335. 7814321 10.1128/jb.177.2.326-335.1995 PMC176595

[mmi13765-bib-0043] Popham, D.L. , and Setlow, P. (1996) Phenotypes of *Bacillus subtilis* mutants lacking multiple class A high‐molecular‐weight penicillin‐binding proteins. J Bacteriol 178: 2079–2085. 8606187 10.1128/jb.178.7.2079-2085.1996 PMC177908

[mmi13765-bib-0044] Rivolta, C. , and Pagni, M. (1999) Genetic and physical maps of the *Bacillus subtilis* chromosome. Genetics 151: 1239–1244. 10101153 10.1093/genetics/151.4.1239 PMC1460559

[mmi13765-bib-0045] Sambrook, J. , Fritsch, E.F. , and Maniatis, T. (1989) Molecular Cloning: A Laboratory Manual. Cold Spring Harbor, NY: Cold Spring Harbor Laboratory Press.

[mmi13765-bib-0046] Sauvage, E. , Derouaux, A. , Fraipont, C. , Joris, M. , Herman, R. , Rocaboy, M. , *et al* (2014) Crystal structure of penicillin‐binding protein 3 (PBP3) from *Escherichia coli* . PLoS One 9: e98042. 24875494 10.1371/journal.pone.0098042 PMC4038516

[mmi13765-bib-0047] Scheffers, D.J. , Jones, L.J. , and Errington, J. (2004) Several distinct localization patterns for penicillin‐binding proteins in *Bacillus subtilis* . Mol Microbiol 51: 749–764. 14731276 10.1046/j.1365-2958.2003.03854.x

[mmi13765-bib-0049] Sobhanifar, S. , King, D.T. , and Strynadka, N.C. (2013) Fortifying the wall: synthesis, regulation and degradation of bacterial peptidoglycan. Curr Opin Struct Biol 23: 695–703. 23910891 10.1016/j.sbi.2013.07.008

[mmi13765-bib-0050] Spratt, B.G. (1975) Distinct penicillin binding proteins involved in the division, elongation, and shape of *Escherichia coli* K12. Proc Natl Acad Sci USA 8: 2999–3003. 10.1073/pnas.72.8.2999 PMC432906 1103132

[mmi13765-bib-0051] Stokes, N.R. , Sievers, J. , Barker, S. , Bennett, J.M. , Brown, D.R. , Collins, I. , *et al* (2005) Novel inhibitors of bacterial cytokinesis identified by a cell‐based antibiotic screening assay. J Biol Chem 280: 39709–39715. 16174771 10.1074/jbc.M506741200

[mmi13765-bib-0052] Valbuena, N. , Letek, M. , Ramos, A. , Ayala, J. , Nakunst, D. , Kalinowski, J. , Mateos, L.M. , and Gil, J.A. (2006) Morphological changes and proteome response of *Corynebacterium glutamicum* to a partial depletion of FtsI. Microbiology 152: 2491–2503. 16849811 10.1099/mic.0.28773-0

[mmi13765-bib-0053] Vavrová, L. , Muchová, K. , and Barák, I. (2010) Comparison of different *Bacillus subtilis* expression systems. Res Microbiol 161: 791–797. 20863884 10.1016/j.resmic.2010.09.004

[mmi13765-bib-0054] Wei, Y. , Havasy, T. , McPherson, D.C. , and Popham, D.L. (2003) Rod shape determination by the *Bacillus subtilis* class B penicillin‐binding proteins encoded by *pbpA* and *pbpH* . J Bacteriol 185: 4717–4726. 12896990 10.1128/JB.185.16.4717-4726.2003 PMC166473

[mmi13765-bib-0055] Wissel, M.C. , Wendt, J.L. , Mitchell, C.J. , and Weiss, D.S. (2005) The transmembrane helix of the *Escherichia coli* division protein FtsI localizes to the septal ring. J Bacteriol 187: 320–328. 15601716 10.1128/JB.187.1.320-328.2005 PMC538840

[mmi13765-bib-0056] Xu, M. (2008) Functional analysis of PBP 2B in *Bacillus subtilis*. Thesis (D. Phil) University of Oxford, Medical Sciences Division.

[mmi13765-bib-0057] Yanouri, A. , Daniel, R.A. , Errington, J. , and Buchanan, C.E. (1993) Cloning and sequencing of the cell division gene *pbpB*, which encodes penicillin‐binding protein 2B in *Bacillus subtilis* . J Bacteriol 175: 7604–7616. 8244929 10.1128/jb.175.23.7604-7616.1993 PMC206916

[mmi13765-bib-0058] Zhao, G. , Meier, T.I. , Kahl, S.D. , Gee, K.R. , and Blaszczak, L.C. (1999) BOCILLIN FL, a sensitive and commercially available reagent for detection of penicillin‐binding proteins. Antimicrob Agents Chemother 43: 1124–1128. 10223924 10.1128/aac.43.5.1124 PMC89121

