# Supplementary material for: Functional redundancy of division specific penicillin‐binding proteins in Bacillus subtilis
Source: Mol Microbiol. 2017 Aug 29;106(2):304–18. doi: 10.1111/mmi.13765 (PMC5656894; doi:10.1111/mmi.13765)
Supplement: Supplementary file 1 — Supporting Figures [file MMI-106-304-s001.pdf]

Supporting information data legend

Fig. S1

Bio-informatic analysis of PBPs

A - Alignment of the proposed active sites of HMT Class 1 PBPs that have defined active sites compared to PBP 2B, SpoVD and PBP 3 of *Bacillus subtilis*. The position of the active site serine residue for PBP 2B and PBP 3 is indicated at the top and bottom of the alignment.

B – Pairwise comparison of PBPs encoded in the *Bacillus* genome. Each box shows the percentage identity and similarity (in parenthesis) for that pair of PBPs along with the region of overlap (including gaps) analysed. The values are shown in red represent PBPs that show significant similarity(> 27.5 % identity) which in most cases, corresponds with PBPs known to have similar functions, if not functional redundancy e.g. PBP 2A and PBP H (Wei *et al*., 2003) and PBP1, PBP 2C, PBP 4 and PBP G (Popham and Setlow, 1996).

Fig S2

Immunofluorescent detection of PBP 3 and PBP 2B

Panel A: A Western blot image of total protein samples of vegetative 168 and 3105 cultures separated by SDS-PAGE using a polyclonal antiserum raised against full length PBP 3. The lanes from left to right correspond to samples of strain 168, and 3105 grown in PAB supplemented with various concentrations of xylose (0 - 0.5 %). The lower arrows corresponds to the position where wild-type PBP 3 would be expected to migrate and the upper arrow for a GFP-PBP 3 fusion.

Panel B: Characteristic images of cells stained for either PBP 3 or PBP 2B using polyclonal antisera that were used to derive “heat map” representations for the subcellular distribution of the PBPs in a cell population relative to cell length. Panels A and B show a typical cell staining patterns obtained for PBP 3 and PBP 2B respectively for the strains 168 and 4001 relative to cell morphology and DNA distribution.

Fig S3.

PBP profiles of strains with conditional expression of *pbpC*.

A - Analysis of oxacillin sensitivity for strains with conditional expression of *pbpC.*

Strains WT (168), ΔpbpC (4015), KS51, KS53, KS52, KS55, 4001, KS51 and KS54 were spotted out as horizontal sequence corresponding to a 1:4 dilution series of the cultures on plates that were incubated for 16 h before being photographed. (A) Nutrient agar alone, (B) Nutrient agar with Oxacillin (0.04μg/ml) and (C) Nutrient agar with oxacillin (0.04 μg/ml) and IPTG (1mM).

B - A fluorogram of an SDS-page gel showing the PBP profile of strains (168, 4001, 4015, KS50, KS51, KS53, KS54, KS52 and KS55 form left to right) sampled in mid-exponential growth in the presence or absence of IPTG (1 mM) and labelled with Bocillin-FL as described in the methods. The relevant genotype of each strain is shown above each lane of the gel. The arrows mark the location of bands corresponding to PBP 2B (1) and PBP 3 (2) that either change in intensity or are absent as expected for the genotype or growth conditions.

Fig. S4.

Aligment of PBP 3, PBP 2B, PBP 2x, PBP 2A

Alignment of PBP 3 to class B PBPs from *S. aureus* (PBP 2A), *B. subtilis* (PBP 2B), *S. pneumonia* (PBP 2X) and *E. coli* (PBP 3) using Uniport database. Sequence similarity was highlighted in three colours blue, red and green ranging from low to high sequence similarity. The N-terminus of PBP 3, which consist of the MecA domain (residues 26-147), showed high similarity only to *Sa*PBP 2A, whereas the dimerization domain (residues 155-322) and the TPase domain (residues 352-666) of PBP 3 showed comparable similarity to all class B PBPs.

Fig. S5

Both PBP 3 and SigM regulated genes act to confer β–lactam resistance. To determine the contributions of *sigM* and *pbpC* to resistance to cell wall synthesis inhibitors exponentially growing cultures of strains 168 (wild type), RD300 (*sigM*), 4015 (*pbpC),* RD301(*pbpC sigM*), and BSU2007 (Δ7 ECF), were serially diluted and spotted onto plates containing moenomycin (A; 8 µg/ml), Penicillin G (B; 0.005 µg/ml), Cephalexin (C; 0.08 µg/ml), Oxacillin (D; 0.04 µg/ml), and no antibiotic (E). Plates were then incubated at 37 °C for 18 h prior to being photographed

Fig. S1

A

```
                                          S309 - PBP 2B
                                            v
gi 239938676 FDPNKRDVTNYYNDLISYAYEPGSTMKIFTLAAAMQENVFNANEKYKSGTFEVGGAPV Pbp2B
gi 239938848 DYQSVDPSVYNRNLPVWSTYEPGSTFKIITLAAALEEQKVNLKRDQFYDKGHAEVDGA SpoVD
2Z2M E       TKEGITEDFVWRDILYQSNYEPGSTMKVMMLAAAIDNNTFPGGEVFNSSELKIADATI Pbp2x S. pneum
gi 75504447  PETGKDFGKKWANDLYQNTYEPGSTFKSYGLAAAIQEGAFDPDKKYKSGHRDIMGSRI Pbp1 S. aureus
gi 83287821  NNLSGTPKEAMRNRTITDVFEPGSTVKPMVVMTALQRGVVRENSVLNTIPYRINGHEI FtsI E. coli
gi 129676    YNKLTEDKKEPLLNKFQITTSPGSTQKILTAMIGLNNKTLDDKTSYKIDGKGWQKDKS MecA S aureus
gi 1175719   WKKLNKDKNNPFSAKFNKTYAPGSTIKPI  AAAIGIKNGTLKADEKKTIKGKEWQKD PbpC
                                            ^
                                          S410 - PBP 3
```

B

**PBP protein**												
**PBP 1A (914aa)**	**PBP 1A**											
**PBP 2A (713aa)**	25.1% (53.3%) 375	**PBP 2A**										
**PBP 2B (716aa)**	24.1% (54.8%) 294	24.3% (54.0%) 643	**PBP 2B**									
**PBP H (685aa)**	-	42.8% (74.7%) 703	23.8% (53.4) 562	**PBP H**								
**PBP 2C (714aa)**	33.2% (65.2%) 742	-	-	-	**PBP 2C**							
**PBP 3 (668aa)**	-	24.4% (54.8%) 610	27.2% (54.9%) 541	-	26.9% (51.6%) 335	**PBP 3**						
**PBP 4 (624aa)**	31.8% (64.0%) 606	-	-	-	33.5% (64.4%) 576	24.6% (50.8%) 382	**PBP 4**					
**PBP 4* (451aa)**	-	-	-	-	-	27.0% (51.3%) 337	-	**PBP 4***				
**PBP G (647aa)**	31.7% (63.3%) 665	-	24.3% (54.2%) 354	22.8% (49.7%) 382	33.8% (66.2%) 625	23.0% (51.8%) 330	30.0% (62.5%) 594	-	**PBP G**			
**SpoVD (645aa)**	-	27.2% (54.2%) 570	33.9% (63.7%) 641	26.5% (56.6%) 449		27.7% (55.9%) 538	23.4% (50.6%) 427	-	22.5% (51.2%) 365	**Spo VD**		
**PBP I (584aa)**	25.4% (53.2%) 327	22.1% (56.6%) 516	24.2% (55.5%) 591	25.0% (55.1%) 508	22.8% (51.8%) 556	23.2 (53.5%) 565	-	-	-	27.9% (55.3%) 591	**PBP I**	
**PBP X (391aa)**	-	-	-	-	-	-	-	31.3% (59.9% 319	-	-	-	**PBP X**

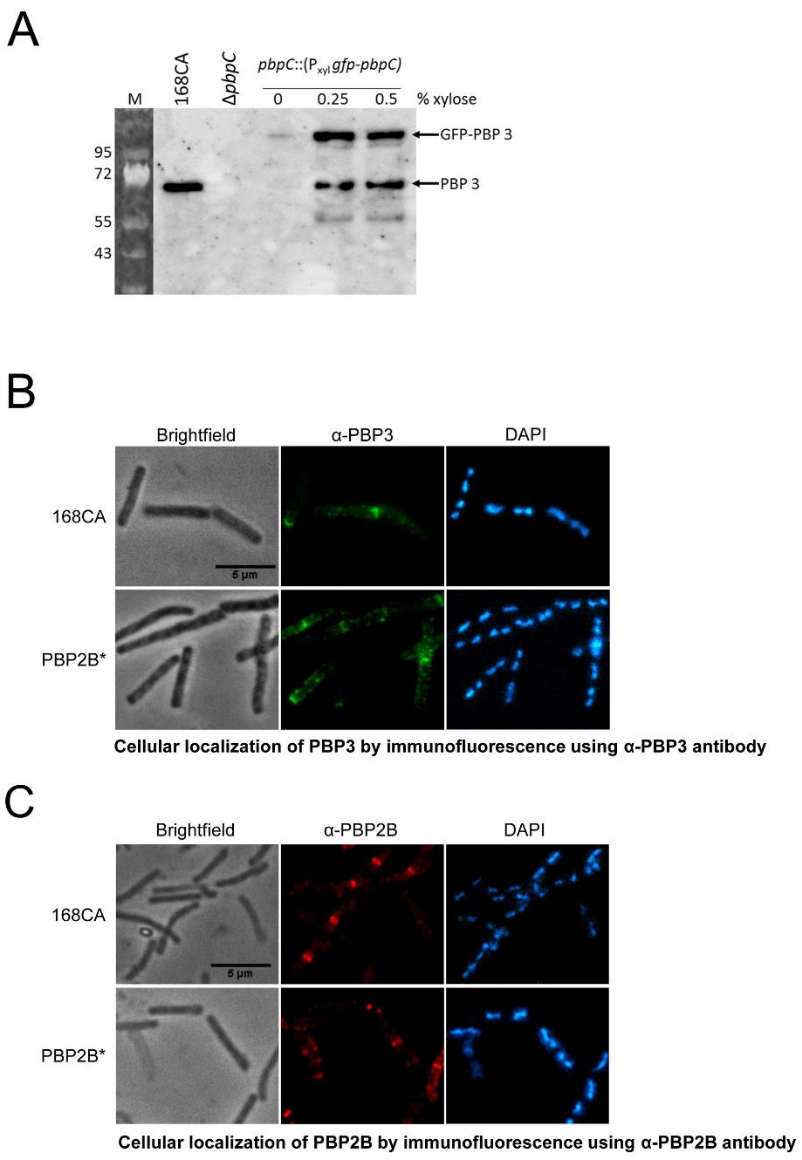
A
M
168CA
ΔpbpC
pbpC::(P xyl gfp-pbpC)
0
0.25
0.5
% xylose
95
72
55
43
GFP-PBP 3
PBP 3
B
Brightfield
α-PBP3
DAPI
168CA
PBP2B*
5 µm
Cellular localization of PBP3 by immunofluorescence using α-PBP3 antibody
C
Brightfield
α-PBP2B
DAPI
168CA
PBP2B*
5 µm
Cellular localization of PBP2B by immunofluorescence using α-PBP2B antibody

Fig S2

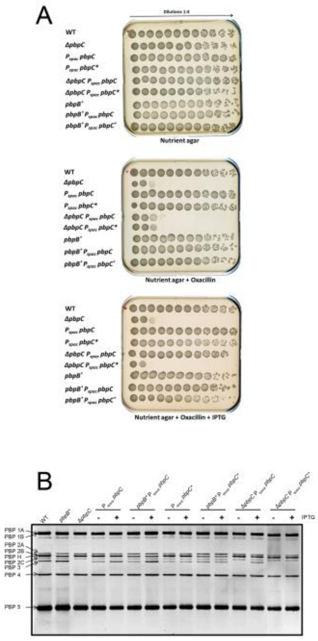
A
Dilutions 1:4
WT
ΔpbpC
Pspac pbpC
Pspac pbpC*
ΔpbpC Pspac pbpC
ΔpbpC Pspac pbpC*
pbpB⁺
pbpB⁺ Pspac pbpC
pbpB⁺ Pspac pbpC⁺
Nutrient agar
Nutrient agar + Oxacillin
Nutrient agar + Oxacillin + IPTG
B
WT
pbpB⁺
ΔpbpC
IPTG
PBP 1A
PBP 1B
PBP 2A
PBP 2B
PBP H
PBP 2C
PBP 3
PBP 4
PBP 5

```
PBPC_BACSU   MLKKCI---LLVFLCVGLIGLIGCSKTDSPEDRMEAFVKQWNDQQFDDMYQSLTKDVKKE 57
O54286_STAAU MKKIKIVPLILIVVVVGFGIYFYA----SKDKEINNTIDAIEDKNFKQVYKDSSYISKSD 56
PBPB_BACSU   ------------------------------------------------------------
PBPX_STRR6   ------------------------------------------------------------
FTSI_ECOLI   ------------------------------------------------------------

PBPC_BACSU   ISKKDFVNRYKAIYEQAGVKNLKVTAGEVDKDDQDNKTMKHIPYKVSMNTNAGKVSFKNT 117
O54286_STAAU NGEVEMTERPIKIYNSLGVKDINIQDRKIKKVSKNKKR-VDAQYKI--KTNYGNID---- 109
PBPB_BACSU   ---------------------------MIQMPKKNK-------F---MN--RGAAILSIC 21
PBPX_STRR6   ---------------------MKWTKRVIRYATKNR----KSPA---ENRRRVGKSLSLL 32
FTSI_ECOLI   ---------------------MKAAAKT-QKPKRQEE---HANF---IS--WRFALLCGC 30

PBPC_BACSU   AVLKLEKTDDEESWNIDWDPS--FIFKQLADDKTVQIMSIEPKRGQIYDKNGKGLAVNTD 175
O54286_STAAU RNVQFNFVKEDGMWKLDWDHS--VIIPGMQKDQSIHIENLKSERGKILDRNNVELANTGT 167
PBPB_BACSU   FALFFFVILGRMAYIQITGKANGEVLATKATEQHEKKRTIEASRGSILDRKGKVIAEDTA 81
PBPX_STRR6   SVFVFAIFLVNFAVIIGTGTRFGTDLAKEAKKVHQTTRTVPAKRGTIYDRNGVPIAEDAT 92
FTSI_ECOLI   ILLALAFLLGRVAWLQVISP---DMLVKEGDMRSLRVQQVSTSRGMITDRSGRPLAVSVP 87

PBPC_BACSU   VPEIGIVPGEL------------GDKKEKVIKELAKKLDLTEDDIKKKLDQGWVKDDSFV 223
O54286_STAAU AYEIGIVPKNV------------SKKD---YKAIAKELSISEDYIKQQMDQNWVQDDTFV 212
PBPB_BACSU   TYKLIAILDKKMTTDVKHPQHVV--NKEKTAEALSKVINLDKADILD-ILNKDAKQVEFG 138
PBPX_STRR6   SYNVYAVIDENYKSATGKILYVEKTQFNKVAEVFHKYLDMEESYVREQLSQPNLKQVSFG 152
FTSI_ECOLI   VKAIWADPKEV--HDAGGISV--GDRWKALANALNIPLD--------QLS----ARINAN 131

PBPC_BACSU   PLKKV------KPDQEKLVSEATSLQGVTRTNVSSRYYPYGEKTAHLTGYVRAITAEELK 277
O54286_STAAU PLKTV------KKMDEYLSDFAK-KFHLTTNETESRNYPLEKATSHLLGYVGPINSEELK 265
PBPB_BACSU   SAGRDITYSQK------QKIEKMKLPGISFLRDTKRYYPNGVFASNLIGYAEVDEE---- 188
PBPX_STRR6   AKGNGITYANM--MSIKKELEAAEVKGIDFTTSPNRSYPNGQFASSFIGLAQLHEN---- 206
FTSI_ECOLI   PKGRFIYLARQVNPDMADYIKKLKLPGIHLREESRRYYPSGEVTAHLIGFTNVDS----- 186

PBPC_BACSU   KKKEGTYSDTSNIGIAGLENVYEDKLRGTTGWKIYVPQT-----GEVI-AEKKAKDGEDL 331
O54286_STAAU QKEYKGYKDDAVIGKKGLEKLYDKKLQHEDGYRVTIVDDNSNTIAHTL-IEKKKKDGKDI 324
PBPB_BACSU   -----T---NEISGAMGLEKVLDKYLKERDGYVTYESDKSGWELPNSKNKITAPKNGDNV 240
PBPX_STRR6   -----EDGSKSLLGTSGMESSLNSILAGTDGIITYEKDRLGNIVPGTEQVSQRTMDGKDV 261
FTSI_ECOLI   ------------QGIEGVEKSFDKWLTGQPGERIVRKDRYGRVIEDISS--TDSQAAHNL 232

PBPC_BACSU   HLTIDIKTQMKLYDELK-------DDSGAAVALQPKTGETLALVSAPSYDPNGFIFGWSD 384
O54286_STAAU QLTIDAKVQKSIYNNMK-------NDYGSGTAIHPQTGELLALVSTPSYDVYPFMYGMSN 377
PBPB_BACSU   YLTIDQKIQTFLEDSMTKVAQKYNPKKIMAAVVDPKTGKVLAMGQRPSFDPNKRDVT--- 297
PBPX_STRR6   YTTISSPLQSFMETQMDAFQEKVKGKYMTATLVSAKTGEILATTQRPTFDADTKEGITED 321
FTSI_ECOLI   ALSIDERLQALVYRELNNAVAFNKAESGSAVLVDVNTGEVLAMANSPSYNPNNLSGTPKE 292

PBPC_BACSU   KEWKKLNKDKNNPFSAKFNKTYAPGSTIKPIAAAIGIKNGTLKADEKKT-----IK---G 436
O54286_STAAU EEYNKLTEDKKEPLLNKFQITTSPGSTQKILTAMIGLNNKTLDDKTSYK-----ID---G 429
PBPB_BACSU   -NYY----------NDLISYAYEPGSTMKIFTLAAAMQENVFNANEKYKSGTFEVGGAPV 346
PBPX_STRR6   FVWR----------DILYQSNYEPGSTMKVMMLAAAIDNNTFPGGEVFNSSELKIADATI 371
FTSI_ECOLI   -AMR----------NRTITDVFEPGSTVKPMVVMTALQRGVVRENSVLNTIPYRINGHEI 341
```

```
PBPC_BACSU   KEWQKDSSWGGYSVTRVSERLQQVDLENALITSDNIYFAQNAL-DMGADTFTKGLKTFGF 495
O54286_STAAU KGWQKDKSWGGYNVTRYEVVNGNIDLKQAIESSDNIFFARVAL-ELGSKKFEKGMKKLGV 488
PBPB_BACSU   KDHNNGVGW---GPT---------TYHDGVLRSSNVAFAKLAKEKLGYDRLNQYLHKFNF 394
PBPX_STRR6   RDWDVNEGLTGGRMM---------TFSQGFAHSSNVGMT-LLEQKMGDATWLDYLNRFKF 421
FTSI_ECOLI   KDVARYSEL---------------TLTGVLQKSSNVGVSKLAL-AMPSSALVDTYSRFGL 385


PBPC_BACSU   SEDVPYEFPIQK-SSIANDKLDSDILLADTGYGQGQMQMSPLHLATAYTPFVDNGDLVKP 554
O54286_STAAU GEDIPSDYPFYN-AQISNKNLDNEILLADSGYGQGEILINPVQILSIYSALENNGNINAP 547
PBPB_BACSU   YQKTGIDLPGEVSSKINFKYE---FDKASTAYGQASA-VTPIQQIQAATAIANDGKMMKP 450
PBPX_STRR6   GVPTRFGLTDEYAGQLPADNI---VNIAQSSFGQGIS-VTQTQMIRAFTAIANDGVMLEP 477
FTSI_ECOLI   GKATNLGLVGERSGLYPQKQRWSDIERATFSFGYGLM-VTPLQLARVYATIGSYGIY-RP 443


PBPC_BACSU   TLIKKDSQTADVWH---------KQVVTKEGAADITKG---------LKGVVEDERGSAY 596
O54286_STAAU HLLKDT--KNKVWK---------KNIISKENINLLTDG---------MQQVVNKTHKEDI 587
PBPB_BACSU   YVIDHIVDPDKDKTIYQNKPESAGTPISASTAKKVRDI---------LGEVVTSKIGTGQ 501
PBPX_STRR6   KFISAIYDPNDQTAR-KSQKEIVGNPVSKDAASLTRTNMVLVGTDPVYGTMYNHST-GKP 535
FTSI_ECOLI   LSITKVDPPV------------PGERVFP--ESIVRTVVHM-------MESVALPGGGGV 482


PBPC_BACSU   QPVVKGITVAGKTGTAELKTSKDDKDGTENG----WFVG-YDYENKDLLVAMMIQNVQDR 651
O54286_STAAU YR--SYANLIGKSGTAELKMK-QGETGRQIG----WFIS-YDKDNPNMMMAINVKDVQDK 639
PBPB_BACSU   AYKIEGFDVAGKTGTAQIA--GKGGYLDGTDNYIFSFMGMAPKDDPELLIYVAVQQPQLK 559
PBPX_STRR6   TVTVPGQNVALKSGTAQIADEKNGGYLVGLTDYIFSAVSMSPAENPDFILYVTVQQPEHY 595
FTSI_ECOLI   KAAIKGYRIAIKTGTAKKVG-PDGRY---INKYIAYTAGVAPASQPRFALVVVINDPQAG 538


PBPC_BACSU   GGSHYVVEKAKKQFQSN------------------------------------------- 668
O54286_STAAU GMASYNAKISGKVYDEL--YENGNKKYDIDE----------------------------- 668
PBPB_BACSU   AGQSSS-DPVSEIFNP--TMKNSLHYLNIEPTEKSDSDKEETKAQTMPDLTDQTVAAAQK 616
PBPX_STRR6   SGIQLG-EFANPILERASAMKDS---LNLQTTAKALEQVSQQSPYPMPSVKDISPGDLAE 651
FTSI_ECOLI   KYY-GG-AVSAPVFGA--IMGGVLRTMNIEPDALTTGDKNEFVINQGEGTGGRS------ 588


PBPC_BACSU   ------------------------------------------------------------
O54286_STAAU ------------------------------------------------------------
PBPB_BACSU   KAKEENLTPIVIGSDVAVKEQYPKADEEVLTNQKVFLKTGGKIKMPDMTGWSRREVLQYG 676
PBPX_STRR6   ELRRNLVQPIVVGTGTKIKNSSAEEGKNLAPNQQVLILSDKAEEVPDMYGWTKETAETLA 711
FTSI_ECOLI   ------------------------------------------------------------


PBPC_BACSU   -----------------------------------------
O54286_STAAU -----------------------------------------
PBPB_BACSU   ELAGIHIEVSGQGYAVSQS-VKKDKEIKDKTVIKVKFKNPD 716
PBPX_STRR6   KWLNIELEFQGSGSTVQKQDVRANTAIKDIKKITLTLGD-- 750
FTSI_ECOLI   -----------------------------------------
```

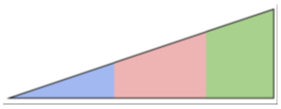

Sequence similarity

Fig S4

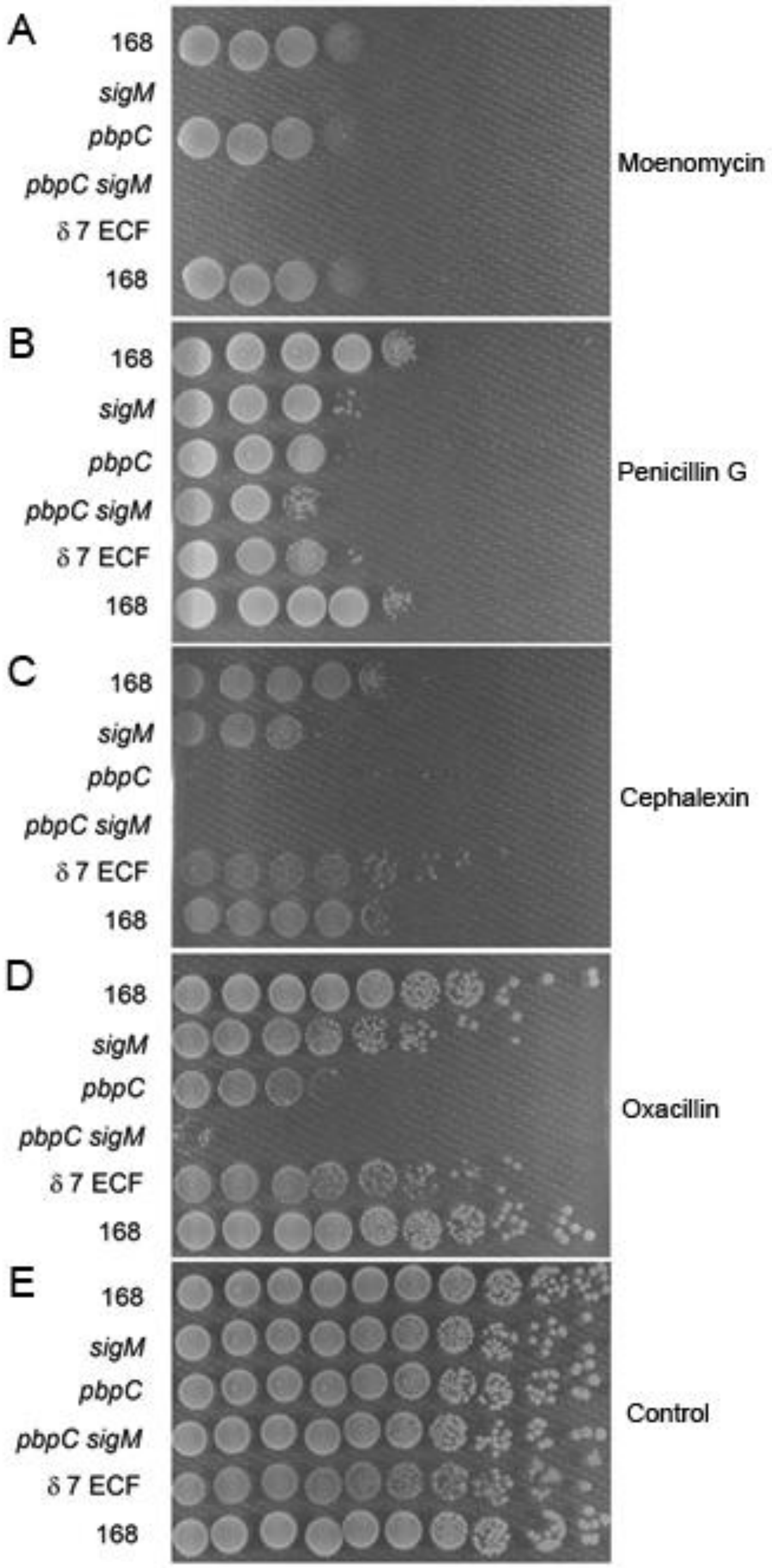
A
168
sigM
pbpC
pbpC sigM
δ 7 ECF
168
Moenomycin
B
168
sigM
pbpC
pbpC sigM
δ 7 ECF
168
Penicillin G
C
168
sigM
pbpC
pbpC sigM
δ 7 ECF
168
Cephalexin
D
168
sigM
pbpC
pbpC sigM
δ 7 ECF
168
Oxacillin
E
168
sigM
pbpC
pbpC sigM
δ 7 ECF
168
Control

Fig S5
